# Dilution Effect for High‐Performance Multiple‐Component Near‐Infrared Organic Photodetectors

**DOI:** 10.1002/advs.202521168

**Published:** 2026-01-20

**Authors:** Zhuang Li, Xunchang Wang, Xiaosong Qiu, Xinpeng Yang, Linming Bi, Jin Chen, Qian Wang, Yurong He, Jingyi Xiong, Renqiang Yang

**Affiliations:** ^1^ School of Optoelectronic Materials and Technology Key Laboratory of Flexible Optoelectronic Materials and Technology (Ministry of Education) Jianghan University Wuhan P. R. China; ^2^ School of Material Science and Engineering Lanzhou Jiaotong University Lanzhou P. R. China

**Keywords:** dilution effect, multiple‐component, near‐infrared organic photodetector, wearable monitoring technology

## Abstract

Despite the demonstrated efficacy of multiple‐component (MC) blends in enhancing the performance of near‐infrared organic photodetector (NIR‐OPD), elucidating the fundamental working mechanism and material design criteria is still crucial for further optimizing MC NIR‐OPDs. Here we elucidate a dilution effect as mechanism in MC‐OPDs featuring two highly miscible constituents. Compared with the binary NIR‐OPD, the MC device with dilution effect enables higher luminescence quantum efficiencies for suppressing non‐radiative decay, significant reduction in dark current density (*J*_d_) to 1.52 × 10^−10^ A cm^−2^ at −0.2 V, and leading to a high detectivity (*D* ^*^) of 6.98 × 10^13^ Jones at 840 nm. Additionally, we revealed that electrons can transfer between different acceptors, governed by their energy level offset, which contributes to the largely unperturbed charge transport and faster response time of 0.63/3.59 µs. Moreover, we successfully validate the efficacy of dilution effect in high‐fidelity pulse signal detection and low‐light NIR imaging, demonstrating great promise for next‐generation wearable health/imaging‐monitoring technologies.

## Graphical abstract

The dilution effect in multi‐component organic photodetector effectively suppresses non‐radiative decay and minimizes dark current, while energy‐level‐offset‐governed electron transfer between acceptors maintains efficient charge transport. This synergistic mechanism collectively enables remarkable device performance, including high detectivity and accelerated response speed, suitable for advanced near‐infrared sensing applications.

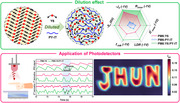

## 1 Introduction

Organic photodetectors (OPDs), renowned for their lightweight nature, flexibility, cost‐effectiveness, and capability for large‐area scaling, have garnered significant attention [1, 2, 3, 4, 5]. Owing to the advancements in material design and synthesis, as well as a variety of device fabrication methods, OPDs can achieve selective spectral response and can be easily adapted for a wide range of applications, such as portable electronics, artificial intelligence systems, automotive technologies, and the Internet of Things [6]. Similar to the case of organic solar cells, construction of multiple‐component (MC) OPD, such as those based on ternary blends, can reduce energetic disorder and prevent unwanted injection from metal contacts, leading to high detectivity (*D* ^*^) values in OPD devices, exceeding 10^14^ Jones [7, 8, 9, 10]. However, considering the largely expanded materials available for the photosensitive active layers, there is still big room for MC‐OPD to achieve high responsivity while minimizing dark and noise currents and ensuring rapid response times via exploring new device engineering [11, 12, 13].

The dilution effect, recognized as an effective strategy for performance optimization in organic optoelectronics such as light‐emitting diodes and solar cells (OSCs), operates on a principle distinct from conventional ternary blending. While conventional approaches primarily focus on optimizing the macroscopic morphology of bulk heterojunctions (e.g., by improving miscibility and reducing domain size) without altering the intrinsic electronic properties of the photoactive materials, the dilution effect aims for molecular‐level modulation. Inspired by the concept of “solid‐state solvation”—analogous to solvatochromism in liquid solutions—this strategy introduces a diluent to achieve molecular‐scale intermixing [14]. Such intermixing disrupts the original molecular packing, enabling precise tuning of key optoelectronic properties including bandgap, charge‐transfer energy, and electron–phonon interactions within the resulting “solid‐state solution,” which ultimately enhances device performance. This effectiveness is evidenced in high‐performance OSCs. For instance, Alex K.‐Y. Jen et al. revealed that the dilution effect effectively modulates a spectrum of optoelectronic properties, inducing blue‐shifted absorption/emission profiles, enhanced luminescence yield, narrowed band tails, and suppressed electron‐phonon coupling, leading to a high performance OSCs over 18% with reduced non‐radiative energy loss [14]. Tao Wang et al. constructed an optimized diluted heterojunction system by incorporating a polymeric diluent, whose extended *π*‐conjugated backbone established interconnected charge‐transport pathways while simultaneously enhancing structural ordering, ultimately achieving a champion PCE of 21% [15]. Despite these empirical successes, a fundamental understanding of the dilution mechanism lags behind, particularly in emerging applications like organic photodetectors (OPDs). Unlike established models such as alloy or planar donor/acceptor models, how dilution specifically affects critical OPD parameters like dark current, noise current and detectivity remains underexplored [16]. As such, the elusive mechanism of the dilution effect hampers the formulation of reliable design rules for device optimization.

In this work, we elucidate the function of dilution effect in MC‐OPDs featuring two highly miscible constituents. Distinct from conventional ternary OPDs which focus on introducing a third component to construct a blended system for either morphology optimization or spectral broadening, the present strategy requires that the third component form a synergistic charge transport network with the donor/acceptor materials [17, 18]. The PY‐IT in this study only acts as a “diluent” which disassembles the excessive crystallization of Y6 without forming an energetically alloyed frontier molecular orbital, while preserving the independent energy levels of each component. As a result, compared with the binary NIR‐OPD, the MC device with dilution effect enables higher luminescence quantum efficiencies for suppressing non‐radiative decay, significant reduction in dark current density (*J*_d_) to 1.52 × 10^−10^ A cm^−2^ at −0.2 V, leading to an ultrahigh *D* ^*^ of 6.98 × 10^13^ Jones. Additionally, we revealed that electrons can transfer between different acceptors, governed by their energy level offset, which contributes to the largely unperturbed charge transport and faster response time of 0.63/3.59 µs. Moreover, we successfully validate the efficacy of dilution effect in high‐fidelity pulse signal detection and low‐light NIR imaging, demonstrating great promise for next‐generation wearable health/imaging‐monitoring technologies.

## 2 Results and Discussion

### 2.1 Dilution Effect

**FIGURE 1:**
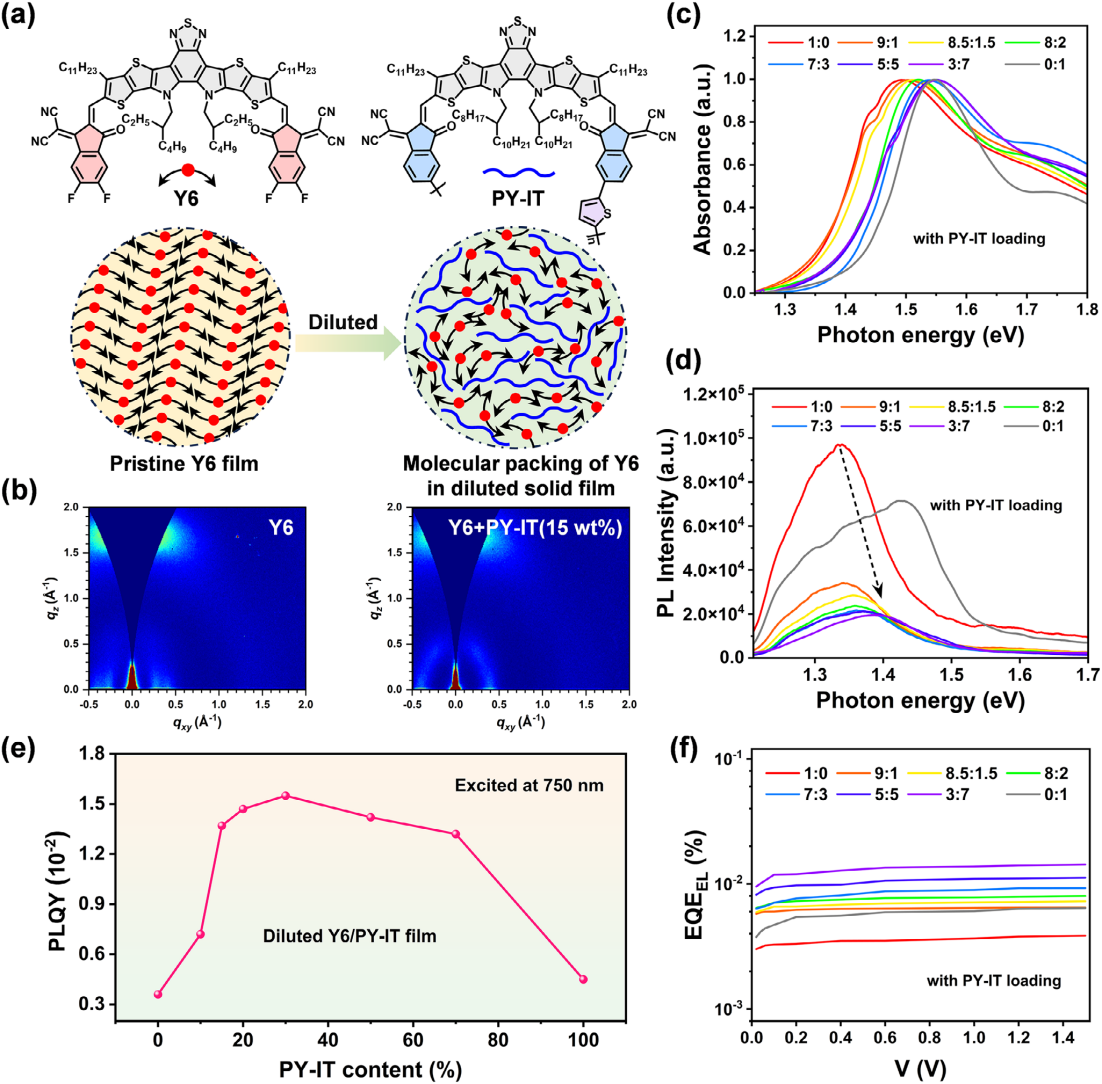
Schematic diagram of dilution effect, molecular structures of Y6 and PY‐IT (a). 2D‐GIWAXS patterns of Y6 and Y6+PY‐IT(15 wt.%) based films (b). Absorption spectra (c) and PL spectra (d) of Y6 blended with PY‐IT at varying weight ratios. The PLQY (e) and EQE_EL_ (f) curves of PM6:Y6 with varying dilution ratios of PY‐IT.

Building upon the “solid‐state solvation” effect observed in organic light‐emitting diodes and the well‐established dilution effect in OSCs, we attempt to investigate its analogous application in MC‐OPDs [19, 20, 21, 22]. This dilution effect, achieved through molecular‐level blending of two distinct components, disrupts the pristine packing order and forms cascaded energy level structure (Figure 1a), while simultaneously inducing key optoelectronic modifications—enlarged bandgap, regulated charge‐transfer energy, and suppressed electron‐phonon coupling—as systematically characterized hereafter [14, 23, 24]. Initially, the blends of Y6:PY‐IT were chosen as our model system. Figure S1 displays the UV‐vis absorption spectra of the pure films. Cyclic voltammetry (CV) measurements were performed to determine the energy levels of the studied materials (Figure S2; Table S2). The polymer acceptor, PY‐IT was selected as the diluent due to its larger bandgap of around 1.40 eV (Table S1) and good miscibility with Y6 (Figure S3; Table S3) [25, 26]. To investigate the morphology changes in the Y6:PY‐IT blend film, we have measured the grazing incidence wide‐angle X‐ray scattering (GIWAXS) spectra of the Y6 pristine film and Y6:PY‐IT blend films with different content of PY‐IT (Figure 1b; Figure S17). The calculated packing parameters are listed in Table S9. Compare to the Y6 pristine film which exhibit distinct OOP (010) peak at *q* = 1.681 Å^−1^ (corresponding to a *d*‐spacing of 3.74 Å) and IP (100) peak at *q* = 0.342 Å^−1^ (*d*‐spacing of 18.36 Å), the incorporation of PY‐IT significantly attenuates the OOP (010) peak intensity. Also, such increase in FWHM of (010) peak indicates a decrease in the CCL as the PY‐IT content increases, reducing from 22.36 Å for pristine Y6 to 22.09 Å for the Y6+PY‐IT blend with 20 wt.% PY‐IT. These results confirm that PY‐IT molecules disrupt the crystalline ordering of Y6, establishing molecular‐scale solubilization or intermixing as the fundamental mechanism underlying the dilution effect [27].

We found that PY‐IT dilution induces a distinct blueshift in both absorption and PL spectra of Y6 films, with the shift magnitude demonstrating clear composition dependence (Figure 1c,d). Additionally, after PY‐IT dilution, the PL is remarkably quenched in Y6:PY‐IT film, with the emission from Y6, which can be attributed to the Förster energy transfer from PY‐IT to Y6 [28]. These results validate the PY‐IT is mixed well with Y6, and the two acceptors exhibits individual function without forming alloyed‐like structures.

The blue‐shift of Y6 emission on mixing is similar to the effect of dissolving Y6 in solvents such as chloroform (Figure S4). In these cases, Y6 aggregation is interrupted by the mediums, causing the optical spectra blue‐shifting [29]. To directly probe the solid‐state energy level shifts, we performed ultraviolet photoelectron spectroscopy (UPS) on the neat and blended films [17]. As shown in Figure S5 and Table S4, The HOMO level of Y6 shows a small but consistent upward shift upon dilution with PY‐IT, with the shift magnitude correlating with the PY‐IT content. This trend aligns perfectly with the observed optical blueshift and confirms a modification of the electronic structure in the solid state. Moreover, the PL quantum efficiency (PLQY) increases gradually by two times on PY‐IT dilution, which is higher than those of PM6:Y6 and PM6:PY‐IT films (Figure 1e). The PLQY results confirm significant suppression of non‐radiative transitions in diluted Y6 domain which might be attributed to inhibited formation of different aggregates (including excimers and other exciplex species), according to the theoretical framework of vibrational coupling [30]. The suppression of non‐radiative transitions can also be verified by external quantum efficiency of electroluminescence that the diluted Y6:PY‐IT system exhibits the higher EQE_EL_ value (Figure 1f) [31, 32]. Meanwhile, after blend with PM6, we found that the diluted ternary blend exhibit sharper at the band tails than PM6:Y6 system (Figure S6), which could lead to the reduced energy disorder in device. As such, we speculated that the diluted effect could be an effect strategy to realize high performance OPDs.

### 2.2 Dilution Effect in MC‐OPD

**FIGURE 2:**
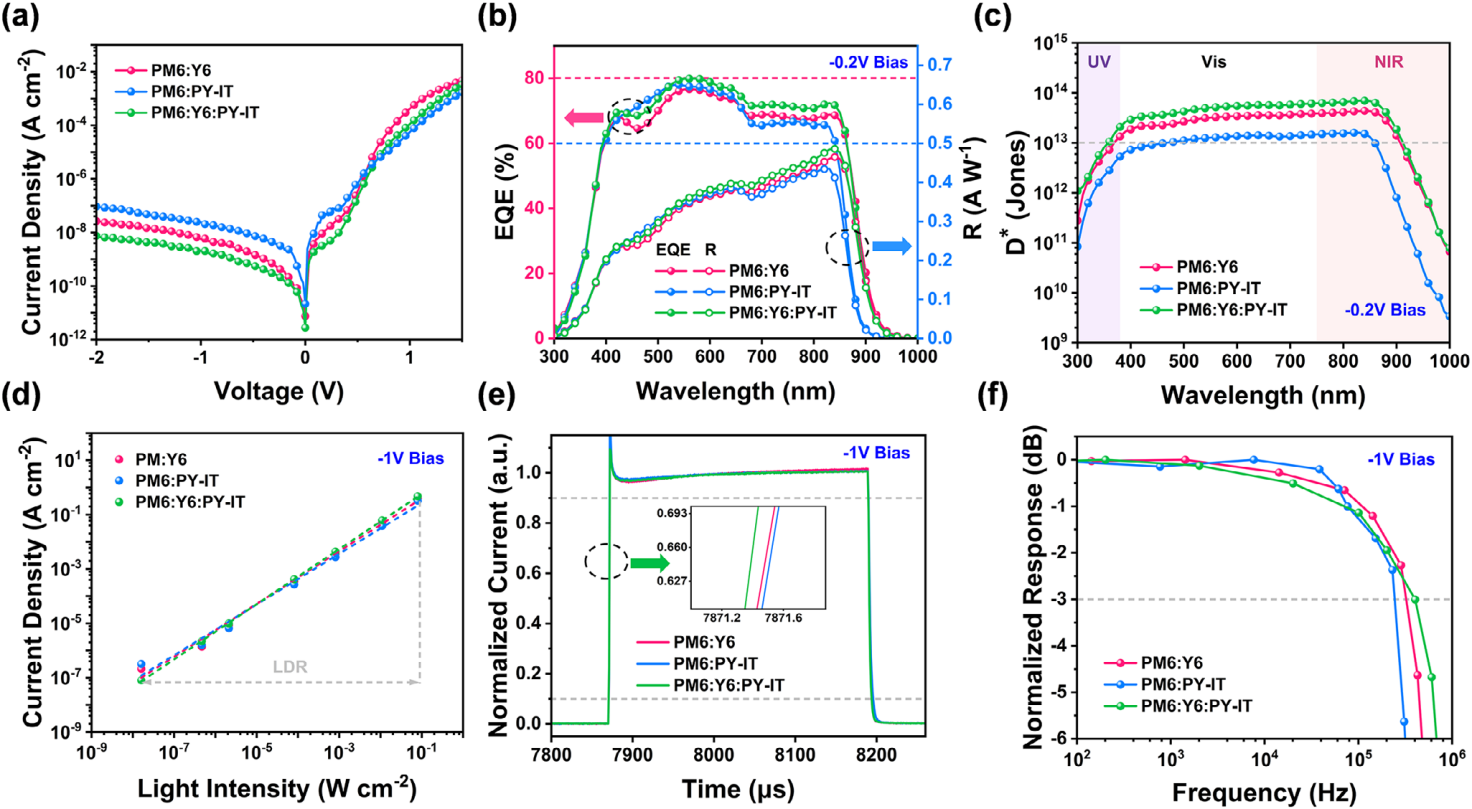
The *J‐V* curves in dark (a). EQE and *R* curves (b), *D* ^*^ curves (c), LDR curves (@ LED 520 nm) (d), rise/fall times (e), and −3 dB cutoff frequency (f) under −0.2 V or −1 V of PM6:Y6, PM6:PY‐IT, and PM6:Y6:PY‐IT(15 wt.%) based OPDs.

Considering the high PLQY value of the diluted Y6:PY‐IT blend (15 wt.% PY‐IT), the diluted system was subsequently incorporated with PM6 polymer donor to fabricate MC‐OPDs, which were compared to the device with non‐diluted blend. From current density–voltage (*J–V*) characteristics of devices, we found that the *J*_d_ under a −0.2 V bias exhibits a non‐monotonic concentration dependence with PY‐IT loading: initially decreasing from 3.51 × 10^−10^ A cm^−2^ (PM6:Y6 non‐diluted device) to 1.52 × 10^−10^ A cm^−2^ (15 wt.% PY‐IT diluted device), then rising to 2.31 × 10^−9^ A cm^−2^ (PM6:PY‐IT), as shown in Figure 2a, Figure S7 and Table 1. This result indicates that diluted device effectively reduces undesirable leakage currents. The reduction could be attributed to the suppression of molecular aggregation and the change of continuous charge transport pathways, which in turn minimizes *J*_d_ and recombination losses under reverse bias [33].

**TABLE 1:** Photodetection performance of PM6:Y6, PM6:PY‐IT and PM6:Y6:PY‐IT(15 wt.%) based devices.

Active layers	*J*_d_ [A cm^−2^] (−0.2 V)	*R*_λ*max*_ [A W^−1^] (−0.2 V)	$D_{\lambda max}^{*}$ [Jones] (−0.2 V)	LDR [dB] (−1 V)	*f*_−3dB_ [kHz] (−1 V)	*t*_rise_/*t*_fall_ [µs] (−1 V)
PM6:Y6	3.51 × 10^−10^	0.465	4.39 × 10^13^	125	324	0.78/3.97
PM6:PY‐IT	2.31 × 10^−9^	0.435	1.60 × 10^13^	120	246	0.86/4.12
PM6:Y6:PY‐IT (15 wt.%)	1.52 × 10^−10^	0.486	6.98 × 10^13^	136	397	0.63/3.59

Then to evaluate the spectral response of the NIR‐OPDs, we measured the EQE and photoresponsivity (*R*) of the above devices under −0.2, −1, and −2 V bias conditions (Figure 2b; Figure S8; Table S5). The overall increase in EQE at 650–850 nm was observed for the diluted devices compared to the two binary devices. Specially, the EQE value of dilute devices at 820 nm at a low bias of −0.2 V is 72.4%, which is higher than PM6:Y6 (68.8%) and PM6:PY‐IT (65.1%). The enhanced EQE can be attributed to the increased exciton dissociation interface in the diluted blend, as well as the enhanced charge transport and carrier collection [34]. The *R*, defined as the ratio of electrical output per optical input in units of A W^−1^ [40], can be calculated by the following equation:

$$R=\frac{\mathrm{EQE}}{100\%}\;\times \;\frac{\lambda }{1240\left(nm\;W\;A^{-1}\right)}$$

where 𝜆 denotes to the wavelength of the incident photon. Apparently, the diluted device (15 wt.% PY‐IT) exhibits a superior *R* of 0.486 A W^−1^ at 840 nm and −0.2 V bias, outperforming both binary devices as summarized in Table 1. Thus, the MC‐OPD with diluted effect exhibits both reduced *J*_d_ and enhanced *R*, contributing to a lower noise level and improved capability in detecting weak optical signals.

To quantify how the dilution effect influence the detection ability of the OPDs [33], Specific detectivity (D^*^) is obtained by the following expression:

$$D^{*}=\;\frac{R\sqrt{A}}{\sqrt{2qI_{d}}}=\frac{R}{\sqrt{2qJ_{d}}}$$

where *q* is the elementary charge (1.6 × 10^−19^ C). Figure 2c displays the calculated D^*^ as a function of wavelength. Notably, the effective suppression of dark current without compromising photocurrent resulted in a higher D^*^ over the entire spectral range (350–950 nm) for the diluted device, measured under a reverse bias of −0.2 V. Specifically, at 840 nm and −0.2 V bias, the diluted device exhibit a calculated D^*^ value of 6.98 × 10^13^ Jones, which is significantly higher than those of binary devices (4.39 × 10^13^ Jones for PM6:Y6 and 1.60 × 10^13^ Jones for PM6:PY‐IT). Remarkably, the diluted devices still remain a D^*^ of exceeding 1.15 × 10^12^ Jones from 860 to 950 nm, ranking among the top NIR detectives in OPDs reported to date (Figure S9; Table S6). Meanwhile, we measured the device's detection performance at −1 and −2 V bias (Figure S8; Table S5), even under a high bias of −2 V, the diluted device still achieves a high D^*^ of 1.06 × 10^13^ Jones, demonstrating the efficiency of dilution effect in improving the NIR detection performance. Regarding the concern about the statistical robustness of device performance, we supplemented the statistical distribution plots of dark current density, *R*, and D^*^ for 20 samples of binary devices and diluted devices under −1 V bias (Figure S10). The device count distribution in each performance interval was presented via histograms, and data dispersion was verified by Gaussian fitting, confirming the statistical reliability of the device performance. Moreover, by replacing the donor material with D18, the NIR detection performances of the D18: Y6: PY‐IT based diluted devices were tested, the key photodetection performance data summarized in Figure S11 and Table S7. The results demonstrate that the D18‐based system also achieves performance improvement after dilution, proving that the dilution design principle could be extended to different donor‐acceptor systems.

In addition to *R* and *D* ^*^, the linear dynamic range (LDR) is another crucial metric for photodetectors [35, 36, 37]. We investigated the light‐intensity‐dependent current–voltage (*J–V*) characteristics of the OPDs, and the LDR was calculated using the following equation:

$$\;\mathrm{LDR}\;=20\log\left(\frac{J_{max}}{J_{min}}\right)\left(dB\right)$$

where *J_max_* is the current feedback under the strongest light intensity, and *J_min_* is the current generated under low light intensity. The photocurrent of the PM6:Y6 and PM6:PY‐IT based binary devices exhibits a linear relationship with light intensity over a range from 0.16 nW cm^−2^ to 80 mW cm^−2^ at a bias voltage of −1 V with irradiation at 520 nm, achieving an LDR of 125 and 120 dB, respectively (Figure 2d). Remarkably, the Y6: PY‐IT diluted device achieves a higher LDR of 136 dB, which can be attributed to its reduced noise current power spectral density (*S_n_*) (Figure S12). Additionally, the transient response of the devices was analyzed by measuring the rise time (*t*_rise_), which is the interval for the signal to increase from 10% to 90% of its maximum, and the fall time (*t*_fall_), which is the interval for the signal to decay from 90% to 10% of its maximum [38]. Notably, the diluted Y6:PY‐IT device demonstrates superior response speeds (*t*_rise_/*t*_fall_ = 0.63 µs/3.59 µs) compared to its binary counterparts (PM6:Y6: 0.78 µs/3.97 µs; PM6:PY‐IT: 0.86 µs/4.12 µs) (Figure 2e). Furthermore, the highest −3 dB cutoff frequency (*f*_−3 dB_) value of 397 kHz for diluted device, echoing its fastest photoresponse and superior charge transport ability (Figure 2f; Figure S13) [39].

### 2.3 Dilution Effect on Energy Disorder and Trap Density

**FIGURE 3:**
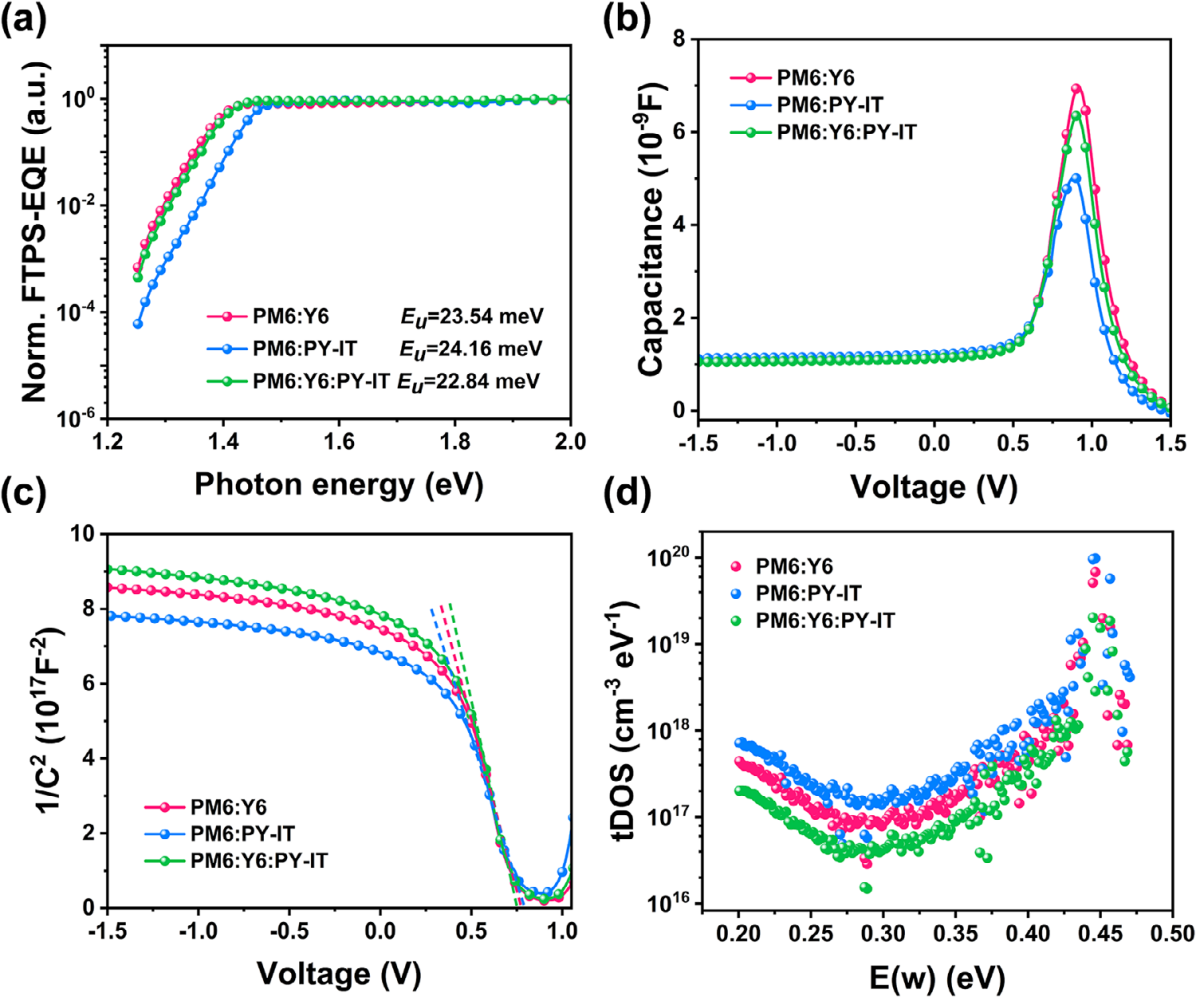
FTPS‐ EQE spectra (a), Capacitance–Voltage (*C‐*‐*V*) curves (b), 1/*C* ^2^‐*V* curves and Mott–Shockley plots (dashed lines represent the linear fitting) (c), *t*DOS plot (d) of PM6:Y6, PM6:PY‐IT, and PM6:Y6:PY‐IT(15 wt.%) based devices.

High‐performance OPDs require low trap densities to minimize recombination, resulting from smaller energetic and structural disorder [40], as reflect by their Urbach energy (*E*_U_). High‐sensitive EQE spectrum revealed an *E*_U_ value of 22.84 meV for the diluted MC‐OPD, lower than both binary systems (23.54 meV for PM6:Y6 and 24.16 meV for PM6:PY‐IT) (Figure 3a). This reduced energy disorder suggests the performance enhancement in MC‐OPDs originates from dilution‐induced trap density reduction in the active layer, which suppresses thermally activated carrier generation and, consequently lowers dark current [41, 42]. To further elucidate the reasons behind the decreased dark current in diluted MC‐OPDs, the capacitance‐voltage (*C*‐*V*) measurements and Mott‐Shockley analysis were performed to investigate the trap density (*N*_A_) and depletion width (*W*) in the devices (Figure 3b) [43]. The *N*_A_ was calculated using the equation:

$$N_{A}=\frac{-2}{q\varepsilon_{r}\varepsilon_{0}A^{2}k}$$

where *q* is the elementary charge, *ɛ*_r_ is the relative dielectric constant as calculated from Figure S14 [44], *ɛ*_0_ is the vacuum permittivity, *A* is the device area (0.04 cm^2^), and *k* is the slope of the linear regime in the 1/*C* ^2^‐*V* curve (Figure 3c). Table 2 summarizes the calculated *N*_A_ values of 1.31 × 10^16^ cm^−3^ (PM6:Y6) and 1.71 × 10^16^ cm^−3^ (PM6:PY‐IT), with the MC‐OPD exhibiting a marginally reduced *N*_A_ of 1.18 × 10^16^ cm^−3^. Meanwhile, the *W* can be estimated according to the equation:

$$W=\sqrt{\frac{2\varepsilon_{r}\varepsilon_{0}\left(V_{bi}-V\right)}{qN_{A}}}$$

where *V* is the applied bias voltage, *V*_bi_ is the built‐in voltage determined from the intersection of the slope line with the voltage axis in Figure 3c. At 0 V bias, the calculated *W* values were 146 nm for PM6:Y6 and 125 nm for PM6:PY‐IT binary OPDs, while the MC‐OPD exhibited a larger value of 155 nm. As the *N*_A_ value showed positive correlation with the *J*_d_ of OPDs [25], therefore, the reduced *N_A_* and increased *W* in diluted MC‐OPDs contribute to their lower *J_d_* characteristics. Furthermore, these variations in *N*_A_ and *W* also provide a consistent explanation for the differing capacitance trends observed across the devices [43]. Moreover, the trap density of states (*t*DOS) of binary and MC‐OPDs were evaluated by the capacitance‐frequency (*C‐ω*) tests [45]. The *t*DOS distribution under varied trap state energy (*E*_ω_) can be extracted according to equation:

$$tDOS\;\left(E_{\omega }\right)=-\frac{\beta V_{bi}}{qAW}\frac{\omega }{k_{B}T}\frac{dC}{d\omega }$$

where 𝛽 is a correction factor, which was set to 1 in this work, *k*_B_ is the Boltzmann constant, and *T* is the absolute temperature. And the *E*_ω_ can be calculated from the equation:

$$E_{\omega }=k_{B}\;Tln\left(\frac{\omega_{0}}{\omega }\right)$$

where *ω*_0_ is the pre‐factor for trap thermal excitation rate, which is usually assumed to be 10^12^ s^−1^ in OPD devices operating in the photovoltaic mode. As shown in Figure 3d, the diluted MC‐OPDs exhibits a lower trap density of 1.48 × 10^16^–2.03 × 10^19^ cm^−3^ eV^−1^ than the PM6:Y6 (2.87 × 10^16^–6.81 × 10^19^ cm^−3^ eV^−1^) and PM6:PY‐IT (4.46 × 10^16^–9.81 × 10^19^ cm^−3^ eV^−1^) at the energy depth of 0.20–0.47 eV. This may facilitate more efficient escape of photo‐generated carriers while suppressing trap‐assisted recombination [40], thereby minimizing photocurrent loss and enhancing EQE in the corresponding OPDs.

**TABLE 2:** The parameters were derived from Mott–Schottky analysis.

Active layers	Slope [F^−2^ V^−1^]	*N*_A_ [cm^−3^]	*V*_bi_ [V]	*W* at 0 V [nm]
PM6:Y6	−2.27 × 10^18^	1.31 × 10^16^	0.77	146
PM6:PY‐IT	−1.87 × 10^18^	1.71 × 10^16^	0.79	125
PM6:Y6:PY‐IT (15 wt.%)	−2.43 × 10^18^	1.18 × 10^16^	0.75	155

The above analyses demonstrate that the dilution strategy induces a critical modification of the solid‐state energy level: it narrows tail states, and lowers *t*DOS and deep trap density of devices. This “sharpening” of the energy profile within the bulk heterojunction is distinct from mechanisms dominated by interfacial dipoles or quadrupole moments to a certain extent which would primarily alter charge injection barriers. In our system, the performance enhancement—specifically the reduction of *J*_d_—is achieved not only by changing the injection barrier but by raising the activation energy for carriers and suppressing trap‐assisted recombination pathways [17, 46].

### 2.4 Dilution Effect on Charge Transport and Recombination

**FIGURE 4:**
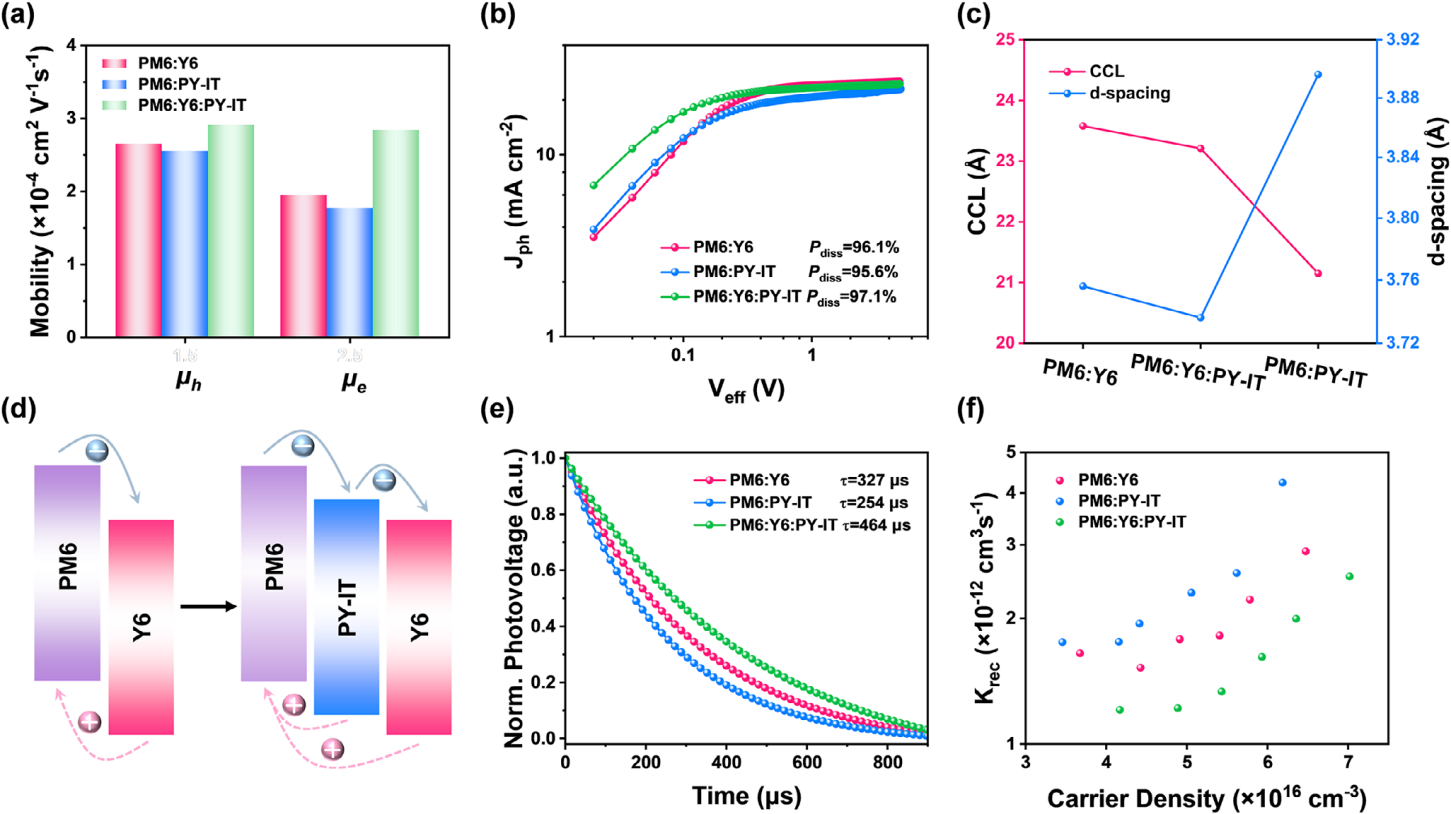
Hole and electron mobility (a). *J*_ph_‐*V*_eff_ characteristics with high reverse bias (b). CCL and d‐spacing curves (c) Schematic diagrams for charge and energy transfer process between PM6 and Y6, and for charge and energy transfer in ternary blends (d), Transient photovoltage (TPV) decays (e) and bimolecular recombination rate constants (*k_rec_*) versus carrier density (f) of PM6:Y6, PM6:PY‐IT, and PM6:Y6:PY‐IT(15 wt.%) devices.

The charge transport properties with dilution effect were systematically investigated using space‐charge‐limited‐current measurements in electron‐only and hole‐only devices (Figure 4a; Figure S15). The dilution process maintained hole mobility at ∼2 × 10^−4^ cm^2^ V^−1^ s^−1^ while remarkably enhancing electron mobility from 1.95 × 10^−4^ to 2.84 × 10^−4^ cm^2^ V^−1^ s^−1^ (Table S8). This improved charge transport behavior reflects the ordered molecular packing observed in the Y6:PY‐IT system (Figures S17 and S18; Table S9), correlating with the reduced trap density and *J*_d_ in diluted OPDs [47]. In addition, the electron‐hole mobility ratio (*µ_e_*/*µ_h_* = 1.02) is much closer to 1, indicating substantially optimized charge transport balance. According to the carrier transit time formula:

$$\tau_{t}=\frac{d^{2}}{\mu V}$$

where *τ_t_* is carrier transit time, *d* is active layer thickness, *µ* is carrier mobility, and *V* is applied bias voltage [48, 49]. With fixed *d* and *V*, higher carrier mobility corresponds to shorter transit time, laying a microscopic foundation for efficient charge extraction. Photocurrent density‐effective voltage analysis (Figure 4b) revealed superior exciton dissociation in PM6:Y6:PY‐IT (15 wt.%) devices (97.1%) compared to the two binary system, indicating more efficient charge transfer at D/A interfaces in the diluted blend. On the basis of the Nyquist plots of three blend films (Figure S16) measured in the frequency range of 20 Hz‐10 MHz under dark conditions, the undiluted PM6:PY‐IT and PM6:Y6 devices exhibit relatively lower recombination resistance (*R_rec_*) of 1.9 and 2.7 MΩ, respectively, which is ascribed to the high density of recombination centers in its active layer [34, 50]. In contrast, the diluted device shows a much higher *R*_rec_ of up to 3.6 MΩ, demonstrating significant suppression of electron‐hole recombination and reduction of charge loss during transport.

To investigate the morphology changes of binary and ternary blend films, atomic force microscopy (AFM), transmission electron microscopy (TEM) and GIWAXS were employed. Figure S18 presents the 2D GIWAXS images and linecut profiles for binary and ternary MC blend films. The calculated packing parameters are listed in Table S9. All blend films display a face‐on dominant orientation, evidenced by the (100) lamellar stacking peak in the in‐plane (IP) direction and the (010) *π‐π* stacking peak along the out‐of‐plane (OOP) direction. Compared with the PM6:Y6 based blend film, the PM6:PY‐IT based blend film exhibits slightly decreased IP (100) peak (from *q* = 0.302 Å^−1^ to *q* = 0.294 Å^−1^) and OOP (010) peak (from *q* = 1.672 Å^−1^ to *q* = 1.612 Å^−1^), indicating the increased lamellar stacking and *π‐π* stacking distance from 20.80 and 3.76 Å for PM6:Y6 to 21.36 and 3.90 Å for PM6:PY‐IT. Accompanied by the significantly decreased crystal coherence lengths (CCLs) for the IP (100) and OOP (010) signals from 72.18 and 23.58 Å for PM6:Y6 to 62.52 and 21.15 Å for PM6:PY‐IT based binary blend film (Figure 4c). this suggests that compare with the PM6:Y6 based blend film, the PM6:PY‐IT based blend film exhibited weaker interchain packing mainly due to the decreased crystallinity of polymer acceptor. After introducing the polymer acceptor into the PM6:Y6 blend film, the OOP (010) peak of the ternary blend film was slightly increased (from *q* = 1.672 Å^−1^ to *q* = 1.681 Å^−1^), indicating a slightly decreased *π‐π* stacking distance of 3.74 Å, and the CCLs for both the IP (100) and OOP (010) signals are also decreased to 69.86 and 23.21 Å, respectively, which is indicative of reduced intermolecular packing. On the basis of the morphology evolution with dilution effect via atomic force microscopy (AFM) and transmission electron microscopy (TEM) measurement, the mutually diluted film showed reduced root‐mean‐square roughness value compared to the binary films. The more uniform phase separation in MC blends (Figure S19) also suggests the aggregation breaking properties with the PY‐IT dilution effect. Moreover, the Y6:PY‐IT blend system with small LUMO offset exhibits a suitable mobility plateau, indicating a facile charge transfer among dilute–diluent domain interfaces (Figure 4d) [51]. Thus, the exciton dissociation enhancement could originate from PY‐IT‐mediated disruption of Y6 aggregation and ideal energy offset, creating additional D/A interfaces that promote charge separation and ultimately boost photo‐response in MC‐OPD.

Furthermore, we found that the charge recombination is suppressed with the dilution effect via analysis of transient photovoltage (TPV) measurement. The PM6:Y6 device exhibited the shortest carrier lifetime (327 µs), which increased to 464 µs upon PY‐IT dilution, indicating less charge recombination (Figure 4e) [52]. This behavior also proved by the evolution of carrier diffusion lengths (*L*_d_) at short circuit based on the equation: $L_{d}=\sqrt{\frac{kT\mu \tau_{c}}{q}}\;$, where *k* is the Boltzmann constant, *T* is the Kelvin temperature, *q* is the elementary charge, 𝜇 is the carrier mobility [53]. After PY‐IT dilution, the *L_d_* values for electrons and holes in the PM6:Y6 blend increased from 406 nm/473 nm to 584 nm/591 nm. Considering that the recombination rate coefficient ($k_{rec}=\frac{1}{(\lambda +1)n\tau }\;$) shows an inverse dependence on carrier density (Figure 4f), where 𝜆 is the recombination order from Figure S20, the PY‐IT diluted system achieved the longest carrier diffusion length and lifetime, yielding the lowest *k_rec_* — direct evidence of recombination suppression via the dilution strategy [54, 55].

### 2.5 The Practical Application of the High‐performance OPD With Dilution Effect

**FIGURE 5:**
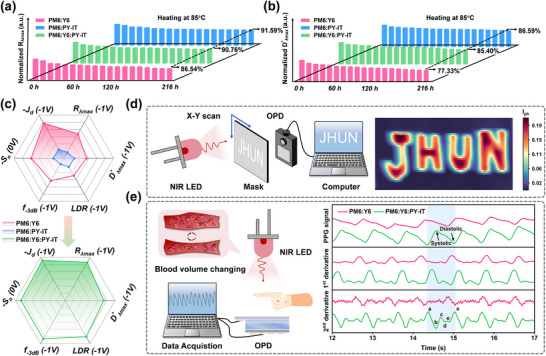
*R*_λ*max*_ (a) and $D_{\lambda max}^{*}$ (b) of unencapsulated OPDs stored in an N_2_‐filled glovebox in the dark while heating at 85°C. Comparison of the optoelectronic performances of binary and optimal ternary device (c). Operating schematic images of NIR single‐point imaging applications and imaging results of “JHUN” pattern under 850 nm LEDs illumination of PM6:Y6:PY‐IT(15 wt.%) devices (d). Schematic diagram of PPG pulse sensors operating, real‐time pulse signal measured by PM6:Y6 and PM6:Y6:PY‐IT(15 wt.%) based OPDs (e).

To assess the practical application of the high‐performance device with dilution effect, the heat stability of these three unencapsulated OPDs were first tested in a nitrogen‐filled glove box while heating at 85°C. As revealed in Figure 5a, Figure S21, and Table S10, after 9 days of heating, the diluted PM6:Y6:PY‐IT (15 wt.%) devices demonstrated exceptional stability, maintaining over 90% of their initial responsivity. This performance is comparable to that of the all‐polymer OPD (where PM6:PY‐IT retained 91.6% of its initial *R* value) and superior to the binary PM6:Y6 OPD (which retained only 86.5% of its initial *R* value). Similarly, under −1 V bias, the maximum detectivity ($D_{\lambda max}^{*}$) for the PM6:Y6‐based device decreased to 6.31 × 10^12^ Jones (Figure 5b). In contrast, the diluted MC‐OPD exhibited a higher and more stable ($D_{\lambda max}^{*}$) of 1.17 × 10^13^ Jones under the same conditions. This enhancement can be attributed to the diluent disrupting continuous charge leakage pathways—thereby stabilizing *J*_d_—and reducing defect and trap density at domain interfaces, which ensures consistent charge collection efficiency and improved detection stability [56, 57].

Through systematic investigation, we demonstrate that the dilution strategy significantly enhances multiple key characteristics of photodetectors, including: *R*, D^*^, LDR, *f*_‐3 dB_, *S_n_*, which displayed in radar map (Figure 5c). These improvements can be attributed to several synergistic factors: (i) the formation of charge transport platforms enabled by diluent incorporation, (ii) ordered molecular packing resulting from disrupted acceptor‐phase aggregation, (iii) reduced deep‐ and shallow‐level trap states, and (iv) substantially suppressed charge recombination dynamics. These findings establish dilution effect as a straightforward yet effective device engineering approach with unique advantages for constructing high‐performance OPDs. Subsequently, we try to implement these optimized MC‐OPD in integrated detecting applications.

Considering the high *R* and D^*^ of the diluted OPD in NIR region, we first conduct the single‐pixel NIR optical imaging under irradiation 850 nm to explore the application promise of obtained OPDs [45, 58, 59]. Figure 5d illustrates the single‐pixel NIR optical imaging setup (40 × 40 resolution, 2 mm step size), featuring a stainless‐steel “JHUN” mask mounted on a motorized XY platform in front of the NIR LED. The photocurrent at 0 V bias was measured using a semiconductor parameter analyzer. When illuminated, the NIR OPD exclusively detects light transmitted through the mask pattern. As shown in Figure 5d, the diluted NIR OPD achieves high‐resolution imaging under 850 nm illumination, accurately reproducing the letter patterns with a clear boundary. In contrast, the non‐diluted device exhibits poor imaging performance at the same incident light intensity. These results indicate the great potential of OPDs with dilution effect for NIR optical imaging applications.

We further demonstrate the application of the NIR OPDs in photoplethysmography‐based heart rate monitoring [36, 60]. This non‐invasive technique utilizes paired NIR LEDs and OPDs positioned on opposite sides of a fingertip. As blood pulses through the capillaries, the resultant fluctuations in blood volume modulate NIR light transmission, which is subsequently converted into photocurrent signals by the OPD. Figure 5e displays real‐time current responses recorded at 850 nm illumination during resting heart rate measurement, with a finger placed between the LED and detector. Compared to binary devices, the diluted MC‐OPD enabling clear differentiation of systolic and diastolic waveforms within each cardiac cycle. This enhanced signal resolution allows accurate heart rate measurement (∼84 bpm, Figure 5e). The improvement aligns with the diluted device's higher detectivity at 850 nm, confirming its advantages for NIR detection requiring deeper tissue penetration.

## 3 Conclusion

In summary, this study elucidates the dilution effect in systems with two highly miscible acceptors, demonstrating its role in enhancing OPD performance. Compared with its binary counterpart, the diluted multi‐component NIR‐OPD exhibits suppressed non‐radiative decay through higher luminescence quantum efficiency, a significantly reduced dark current density of 1.52 × 10^−10^ A cm^−^^2^ at −0.2 V, and a remarkable D^*^ of 6.98 × 10^13^ Jones at 840 nm. By mitigating the severe decline in D^*^ beyond 900 nm—caused by non‐radiative recombination and dark current noise—our diluted device achieves a D^*^ value higher than that of most reported NIR OPDs. The device with dilution effect also exhibits a faster response, demonstrating rise/fall times of 0.63/3.59 µs at −1 V, overcoming the limited response speed typically observed in conventional NIR OPDs at high frequencies. Furthermore, the diluted device demonstrates outstanding performance for real‐time applications—including high‐fidelity pulse monitoring and low‐light imaging—underscoring their strong potential for next‐generation wearable health monitoring and optical sensing technologies.

## Conflicts of Interest

The authors declare no conflicts of interest.

## Supporting information




**Supporting File**: advs73763‐sup‐0001‐SuppMat.docx.

## Data Availability

The data that support the findings of this study are available in the supplementary material of this article.
