# Supplementary material for: Dilution Effect for High‐Performance Multiple‐Component Near‐Infrared Organic Photodetectors
Source: Adv Sci (Weinh). 2026 Jan 20;13(16):e21168. doi: 10.1002/advs.202521168 (PMC13042476; doi:10.1002/advs.202521168)
Supplement: Supplementary file 1 — Supporting File: advs73763‐sup‐0001‐SuppMat.docx. [file ADVS-13-e21168-s001.docx]

**Supporting Information for**

**Dilution effect for high-performance multiple-component** **near-infrared organic photodetectors**

Zhuang Li, Xunchang Wang^*^, Xiaosong Qiu, Xinpeng Yang, Linming Bi, Jin Chen, Qian Wang^*^, Yurong He, Jingyi Xiong, Renqiang Yang^*^

Z. Li, X. Wang, X. Qiu, J. Chen, Y. He, J. Xiong, J. Xiong, R. Yang

Key Laboratory of Flexible Optoelectronic Materials and Technology (Ministry of Education), School of Optoelectronic Materials & Technology, Jianghan University, Wuhan 430056, China

E-mail: wangxc@jhun.edu.cn, [yangrq@jhun.edu.cn](mailto:yangrq@jhun.edu.cn)

X. Yang, L. Bi, Q. Wang

School of Material Science and Engineering, Lanzhou Jiaotong University, Lanzhou 730070, PR China

Email: WQ739424083@163.com

**Experimental Section**

**1. Materials**

All the reagents, unless otherwise specified, were purchased from Sigma-Aldrich Co., J&K, and Tokyo Chemical Industry Co., Ltd., and were used without further purification. PM6, Y6 and PY-IT was purchased from Solarmer Materials Inc.

**2. Method**

**Ultraviolet-Visible absorption spectra (UV-vis):** The UV-vis absorption spectra of solutions and films were recorded using a Hitachi U-4100 spectrophotometer.

**Cyclic voltammetry (CV):** Cyclic voltammetry measurements were carried out on a CHI660A electrochemical workstation with a three-electrode configuration, using Ag/AgCl as the reference electrode, a platinum plate as the counter electrode, and a glassy carbon as the working electrode. Tetrabutylammonium hexafluorophosphate in anhydrous acetonitrile (0.1 mol L^–1^) was used as the supporting electrolyte. A ferrocene/ferrocenium (Fc/Fc^+^) redox couple was used as internal standard and was assigned an absolute energy of -4.8 eV versus vacuum. The HOMO and LUMO energy levels of the materials were determined according to the equation E_HOMO/LUMO_ = -(E_ox_ _onset_/E_red_ _onset_ -E_1/2_^(Fc/Fc+)^ + 4.80).

**Ultraviolet photoelectron spectroscopy (UPS):**UPS spectra were measured by a Kratos Axis Ultra DLD spectrometer. The UPS measurement was performed using a HeⅠ (*hv* = 21.22 eV) source, during which the vacuum of the analysis chamber is maintained at 3.0×10^-8^ Torr and the applied bias voltage is -5 V. The work function (WF), the highest occupied molecular orbitals (HOMO) and the lowest unoccupied molecular orbitals (LUMO) energy levels can be separately obtained according to the following equations:

$$WF=h\nu-E_{cutoff}$$

$$E_{HOMO}=h\nu-{(E}_{cutoff}-E_{onset})$$

$$E_{LUMO}=E_{HOMO}+E_{g}^{opt}$$

where *hv* is the incident photon energy, equal to 21.22 eV; *E_cutoff_* and *E_onset_* are the high binding energy cutoff and the low binding energy onset relative to the [Fermi level](https://www.sciencedirect.com/topics/engineering/fermi-level) (*E_F_*) of substrate, respectively, determined by the tangent extrapolation.

**Grazing incidence wide-angle X-ray scattering characterization (GIWAXS):** 2D-GIWAXS experiments were carried out on a GANESHA 300XL+ system from JJ X-ray. The instrument is equipped with a Pilatus 300 K detector, with pixel size of 172×172 μm. The X-ray source is a Genix 3D Microfocus Sealed Tube X-Ray Cu-source with integrated Monochromator (30 W). The wavelength used is λ = 1.5418 Å. The detector moves in a vacuum chamber with sample-to-detector distance varied between 0.115 m and 1.47 m depending on the configuration used, as calibrated using silver behenate (d001 = 58.380 Å). The minimized background scattering plus high-performance detector allows for a detectable q-range varying from 3×10^−3^ to 3 Å^−1^ (0.2 to 210 nm). The sample was placed vertically on the goniometer and tilted to a glancing angle of 0.2° with respect to the incoming beam. A small beam was used to get a better resolution. The accumulation time was 30 minutes for each measurement. In-plane and out-of-plane line-cuts were obtained using SAXSGUI program.

**Atomic force microscopy (AFM):** Standard tapping-mode AFM measurements in ambient were performed on a Scanned Probe Imaging and Development (SPID) on Park NX10. The AFM images were confirmed from different samples and scan areas. The root-meansquare roughness (RMS) values of the height images were obtained from the whole scan area (2 μm × 2 μm). All the AFM images were flattened and exported from the software.

**Transmission electron microscopy (TEM):** TEM measurements were performed by using a HITACHI H-7650 electron microscope with an acceleration voltage of 120 kV.

**The calculation of Contact angles:** Contact angles were measured with a contact angle meter (GBX DIGIDROP). The solution of each organic material was spin-coated on cleaned ITO substrates. Droplets of H_2_O and CH_2_I_2_ were dripped onto the different films. According to Owens-Wendt method, surface tension could be divided into dispersive and polar components:

𝛾= 𝛾^𝑑^ + 𝛾^𝑝^

Furthermore, the dispersive and polar surface tension can be calculated through the formula below based on the contact angles obtained by two solvents.

(1 + cos 𝜃)𝛾_𝐿_ = $2\sqrt{\gamma_{S}^{d}}\gamma_{L}^{d}$ + $2\sqrt{\gamma_{S}^{P}}\gamma_{L}^{P}$

where 𝜃 is the contact angle of a specific solvent, 𝛾_𝐿_ is the surface tension of the solvent, $\gamma_{S}^{d}$and$\gamma_{L}^{d}$refer to the dispersive and polar surface tension of the solid, respectively. $\gamma_{S}^{P}$and $\gamma_{L}^{P}$refer to the dispersive and polar surface tension of the solvent, respectively. Thus, the unknown values $\gamma_{S}^{d}$and $\gamma_{L}^{P}$can be solved through combining two equations obtained by contact angle measurement of two different solvents.

Calculation of Flory-Huggins interaction parameter (χ) by contact angle Solubility parameter (𝛿) can be calculated from the surface tension:

𝛿 = K$\sqrt{\gamma}$

where γ is the surface tension, K is the proportionality constant (K =116×10^3^ m^-1/2^). Flory–Huggins interaction parameter (𝜒_𝑖𝑗_) can be written as a function of two solubility parameter:

𝑥_𝑖𝑗_ =$\frac{V_{0}}{RT}$𝑉 (𝛿_𝑖_ ‒ 𝛿_𝑗_)^2^

where 𝜒_𝑖𝑗_ is the Flory–Huggins interaction parameter between the material i and j, 𝑉_0_ is the geometric mean of the polymer segment molar volume, R is the gas constant, T is the absolute temperature, and 𝛿_𝑖_ and 𝛿_𝑗_ are the solubility parameter of material i and j, respectively. To simplify, we define the parameter

*κ = K^2^V_0_/RT*

then the Flory–Huggins interaction parameter can be written as the formula below,

𝑥_𝑖𝑗_ = 𝑘( 𝛾𝑖 ‒ 𝛾𝑗)^2^

where 𝛾_𝑖_ and 𝛾_𝑗_ are the surface tension of material i and j, respectively.

**3. Device fabrication and characterization**

The OPD devices were fabricated with the structures ITO/ZnO/BHJ/MoO_3_/Ag. The ITO coated glass substrates were sonicated successively with detergent, deionized water, acetone and isopropanol, and dried with nitrogen flow. Immediately prior to device fabrication, the substrates were cleaned by oxygen plasma for 10 min. The sol–gel ZnO precursor solution was passed through a 0.45-µm filter and deposited onto ITO through spincoating (4000 rpm) in air. The sample was then dried at 200 °C for 1 h and were transferred into the nitrogen-filled glove box to fabricate the photoactive layers. All active layer solutions were prepared with a donor-to-acceptor weight ratio of 1:1.2, with the donor concentration at 12 mg/mL in chloroform. 1-Chloronaphthalene (1-CN) was added as a liquid additive. Ternary devices were fabricated by adjusting the weight ratio of PY-IT in the acceptors. The active layer solutions were spin-coated onto the ZnO layer at different spinning speeds, forming an active layer approximately 200 nm thick. Subsequently, the PM6:Y6 and ternary systems were annealed at 85 °C for 5 minutes, while the PM6:PY-IT system was annealed at 95 °C for 5 minutes. Finally, MoO_3_ (10 nm) and Ag (100 nm) layers were deposited by vacuum thermal evaporation under a vacuum condition of 5×10⁻⁴ Pa.

**Characterization of device:** The current-voltage (*J*-*V*) characteristics were measured with a Keithley 2450 source measurement unit. The OPDs were measured under an irradiation intensity of 100 mW cm^-2^ (AM 1.5 G) by a Newport solar simulator.

**External quantum efficiencies (EQEs):** The EQE spectra were analyzed using an integrated system (LST-QE).

**Fabrication and characterization of single-carrier devices:** The hole and electron mobilities were calculated using the space charge limited current (SCLC) model with a device configuration of ITO/PEDOT:PSS/active layer/MoO_3_/Ag and ITO/ZnO/active layer/ PNDINN/Ag, respectively, where the current density is calculated by:

*J* = 9*ε*_o_ε_r_*µV*^2^/(8*L*^3^)

where *J* stands for current density, ε_o_ is the permittivity of free space, ε_r_ is the relative dielectric constant of the transport medium, *µ* is the hole mobility, *V* is the voltage drop across the device (*V* = *V*_appl_- *V*_bi_ - *V*_RS_, where *V*_appl_ is the applied voltage to the device, *V*_bi_ is the built-in voltage due to the difference in work function of the two electrodes, and V_RS_ is the voltage drop due to series resistance across the electrodes), and *L* is the thickness of the active layer.

**Dielectric constant measurements:** The Dielectric constants were measured with a simple device structure of ITO/ZnO/test film/MoO_3_/Ag at difference frequency from 20 Hz to 2 MHz. According to the previous work, the dielectric constant should be evaluated at the material's geometric capacitance, which represents the capacitance measured when the capacitance derives from only the material itself—the electronic, atomic, and ionic polarization. The stable capacitive response with respect to frequency ranging from 100 kHZ to 1000 kHz is obtained. Relative dielectric constant (*ε_r_*) can be calculated by the equation:

$$\varepsilon_{r}=\frac{C_{P}d}{\varepsilon_{0}A}$$

where *C_p_* is the measured capacitance, *d* is the thickness of film, *A* is the contact area and *ε_0_* is the permittivity of free space.

**Light intensity dependence measurements for *J*_sc_ and *V*_oc_:** The dependence of *J*_sc_ on the light intensities (*P*_light_) follows the power-law $(J_{\mathrm{sc}}\propto\left( P_{\mathrm{light}} \right)^{\alpha})$, where α is the exponential factor. Weak bimolecular recombination in the active layers would result in a linear relationship between *J*_sc_ and *P*_light_ with α close to 1. The relationship of *V*_oc_ dependence on *P*_light_ determines the degree of trap-assisted recombination, which can be evaluated using the formula: $V_{\mathrm{oc}}\propto n\left( kT/q \right)ln(P_{\mathrm{light}})$, where *k* is the Boltzmann constant, *T* is the absolute temperature, *q* is the elementary charge, and $n$ is the ideality factor under illumination and presents the recombination dynamics in the device. The slope of the *V*_oc_-*P*_light_ curve could be higher than *kT/q* when the trap-assisted recombination is involved.

**TPC, TPV and CE measurements:** Relevant control and OPDs were excited with a 522 nm laser diode (repetition frequency: 1 kHz). The transient photocurrent response of the devices at a short circuit condition to a 200 𝜇s square pulse from the LED with no background illumination. The current traces were recorded on a Tektronix DPO3034 digital oscilloscope by measuring the voltage drop over a 5-ohm sensor resistor in series with the OPD. DC voltage was applied to the OPD with an MRF544 bipolar junction transistor in a common collector amplifier configuration. TPV measurement was measured under open-circuit voltage condition, and variable bias light intensities leaded to the change of *V*_OC_. As for CE measurement, firstly, devices were at open circuit and under light, then the light was turn off, the voltage was set to zero or taken to short-circuit condition within a few hundred nanoseconds to extract charges.

**Impedance Spectroscopy measurements:** Impedance Spectroscopy measurements: Impedance spectroscopy was performed using an E4990A impedance analyzer (Keysight Technologies) under dark conditions. An AC perturbation voltage of 50 mV was applied to ensure measurements remained within the linear response regime, with a frequency sweep range from 20 Hz to 10 MHz. All measurements were conducted at room temperature in ambient atmosphere. The obtained impedance data were fitted using Zview software to extract equivalent circuit parameters.

**Thermal admittance spectroscopy (TAS) measurements:** Trap density of state (*tDOS*) was performed on Semiconductor Parameter Analyzer FS338 and analyzed by using TAS method under angular frequency-dependent capacitance measurement (20 Hz to 2 MHz).

**Imaging measurements:** The OPDs imaging experiment used the LBIC photocurrent mapping system (LST-LBIC, Lightsky Technology Co., Ltd), with resolution 40×40, and step size of 2 mm.

**4. Additional figures and tables**

**
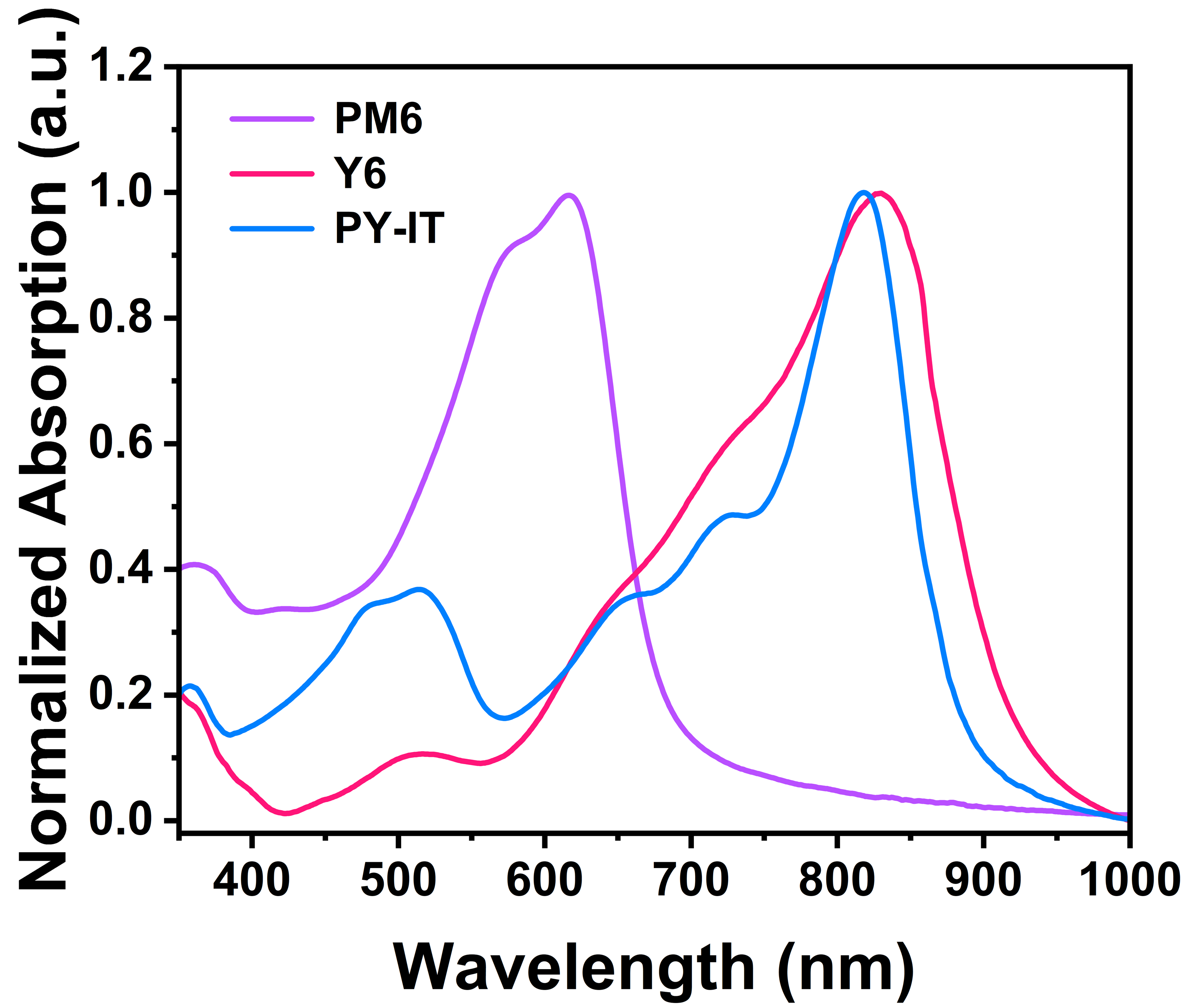
**

**Figure S1.** Normalized UV-vis absorbance spectra of PM6, Y6, PY-IT films.


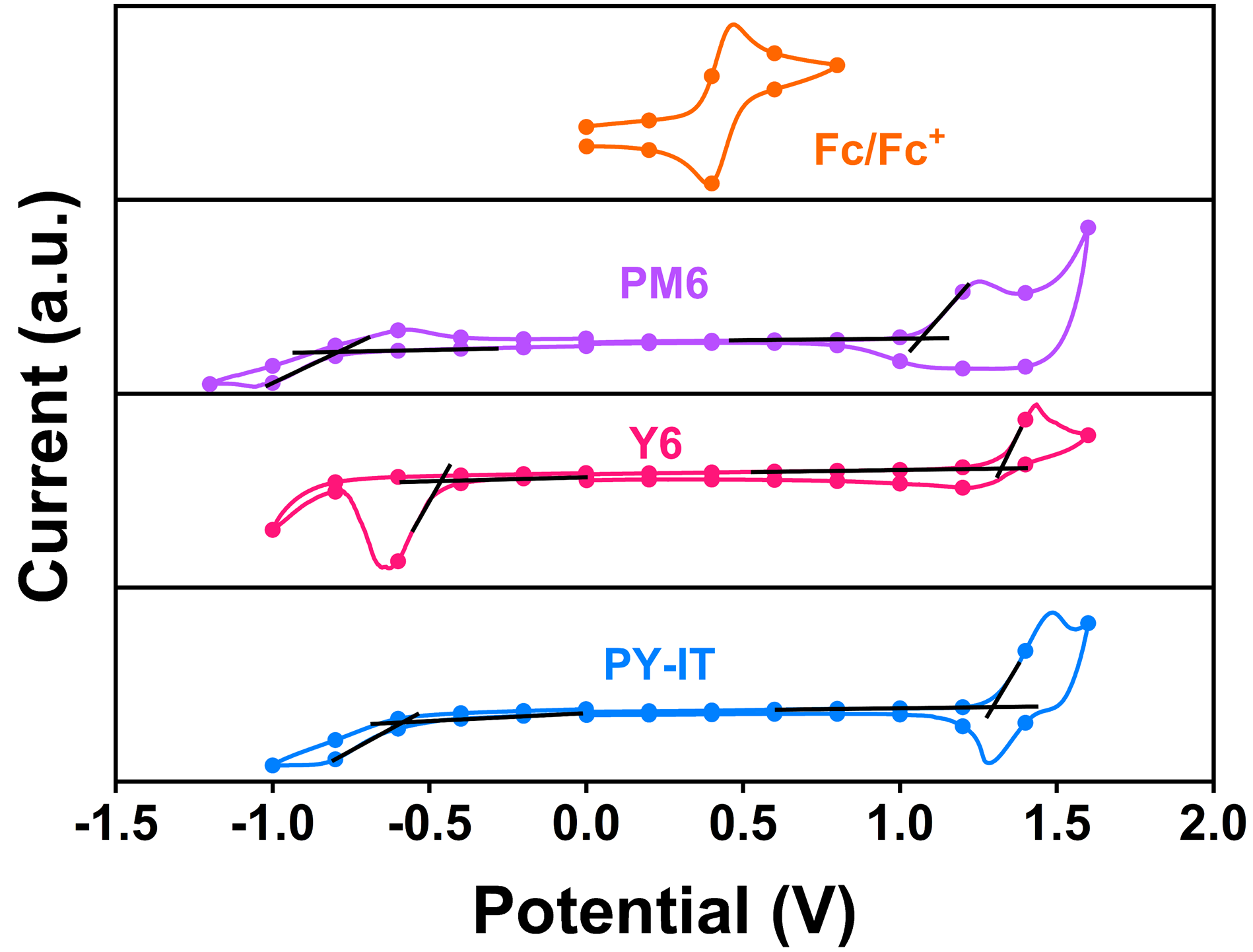


**Figure S2.** Electrochemical cyclic voltammogram curves of PM6, Y6 and PY-IT.


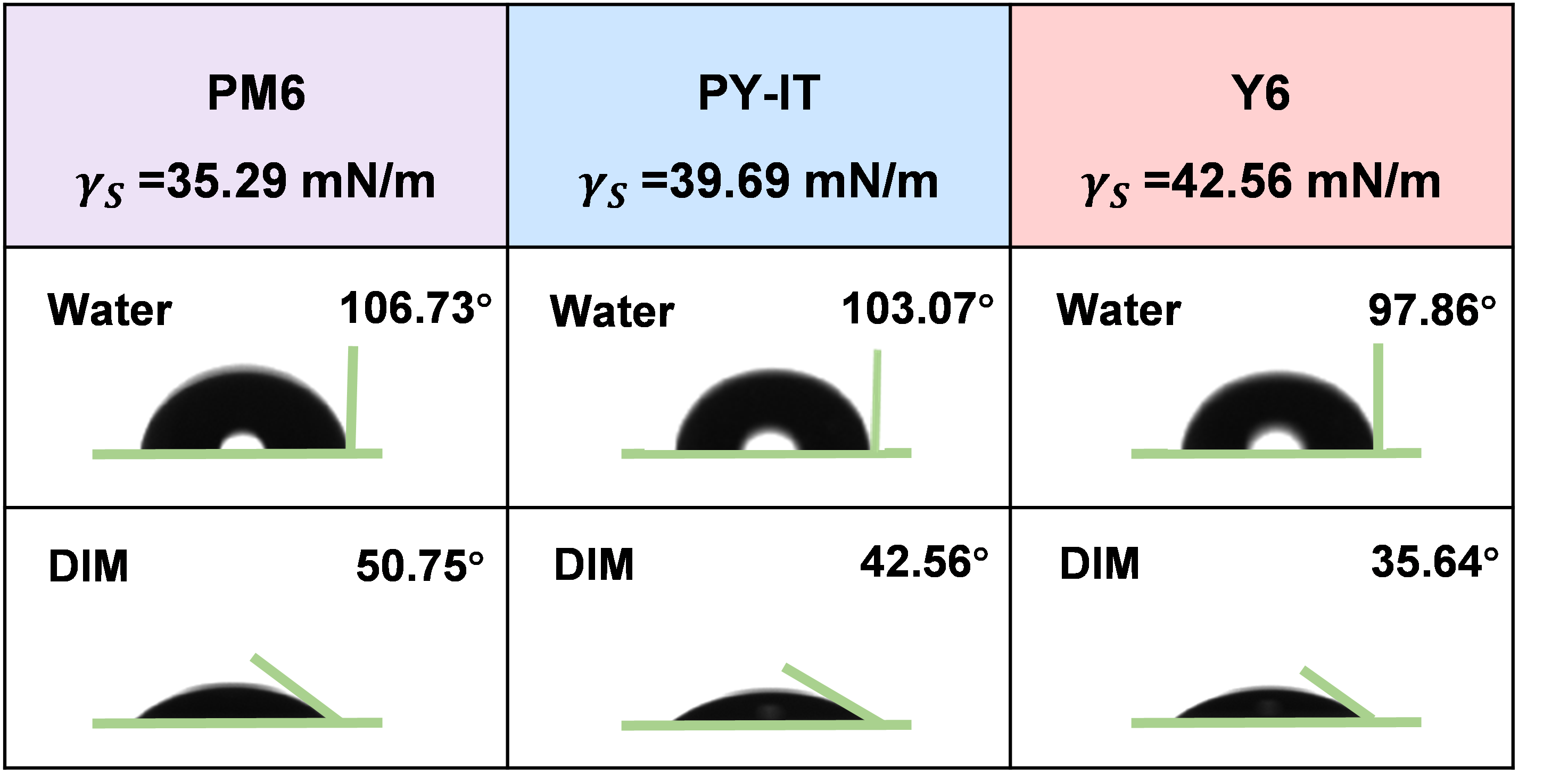


**Figure S3.** Contact angles images of pristine PM6, Y6 and PY-IT films.


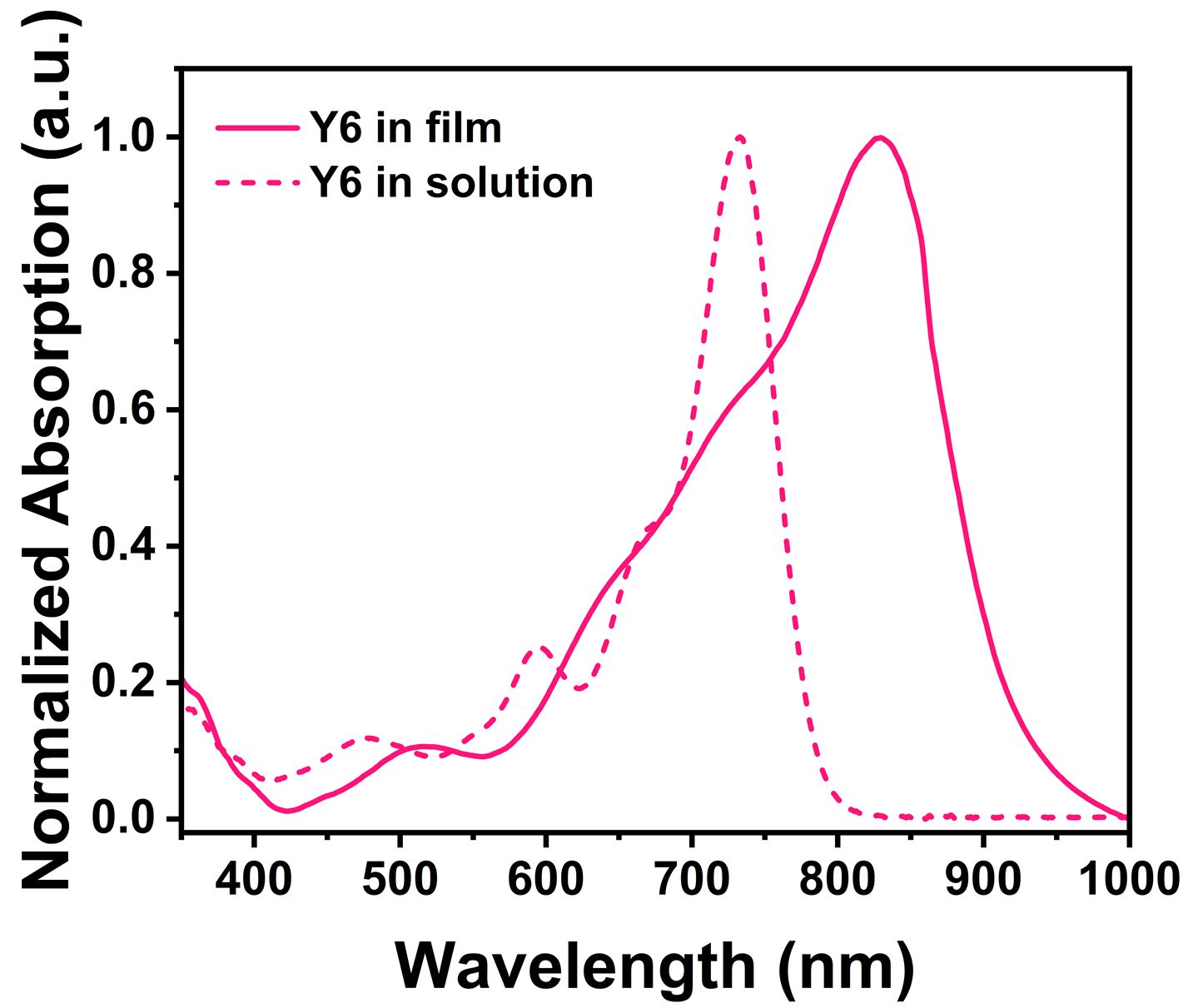


**Figure S4.** Normalized UV-vis absorbance spectra of the Y6 in film and in CHCl_3_ solutions.


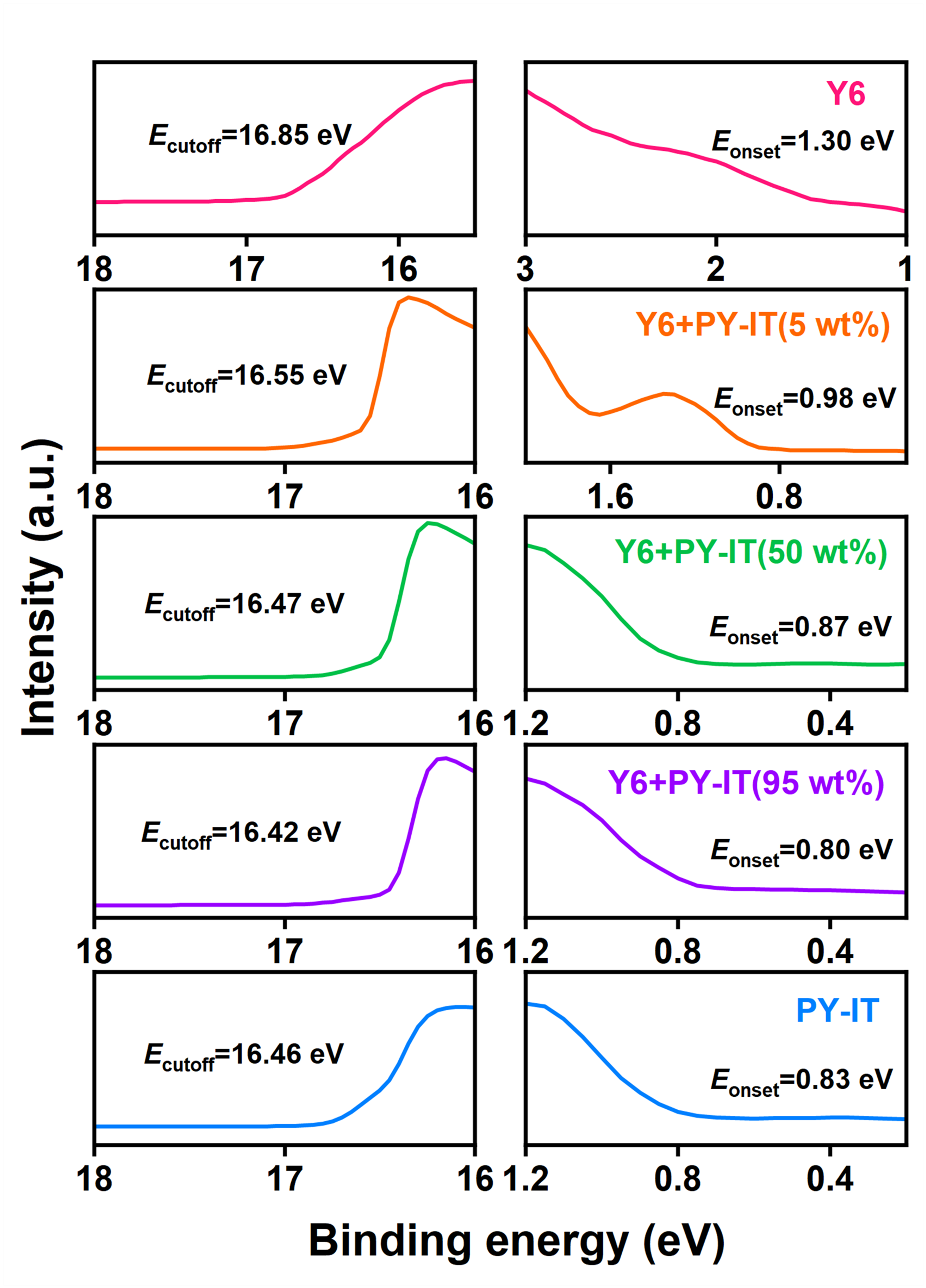


**Figure S5.** UPS measurement of Y6, PY-IT films and Y6 blended with PY-IT at varying weight ratios.


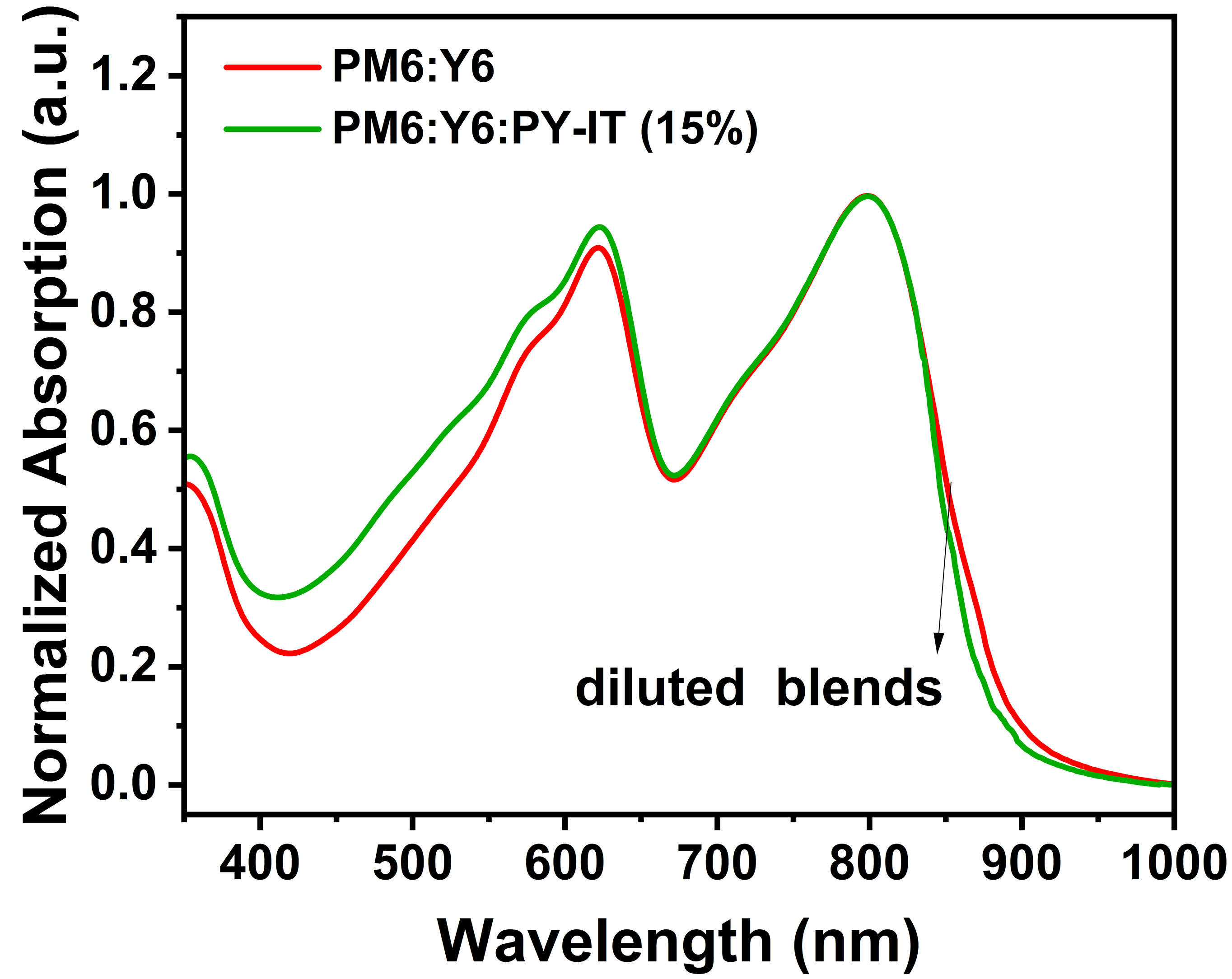


**Figure S6.** Absorption spectra of PM6:Y6 binary and diluted ternary blends.


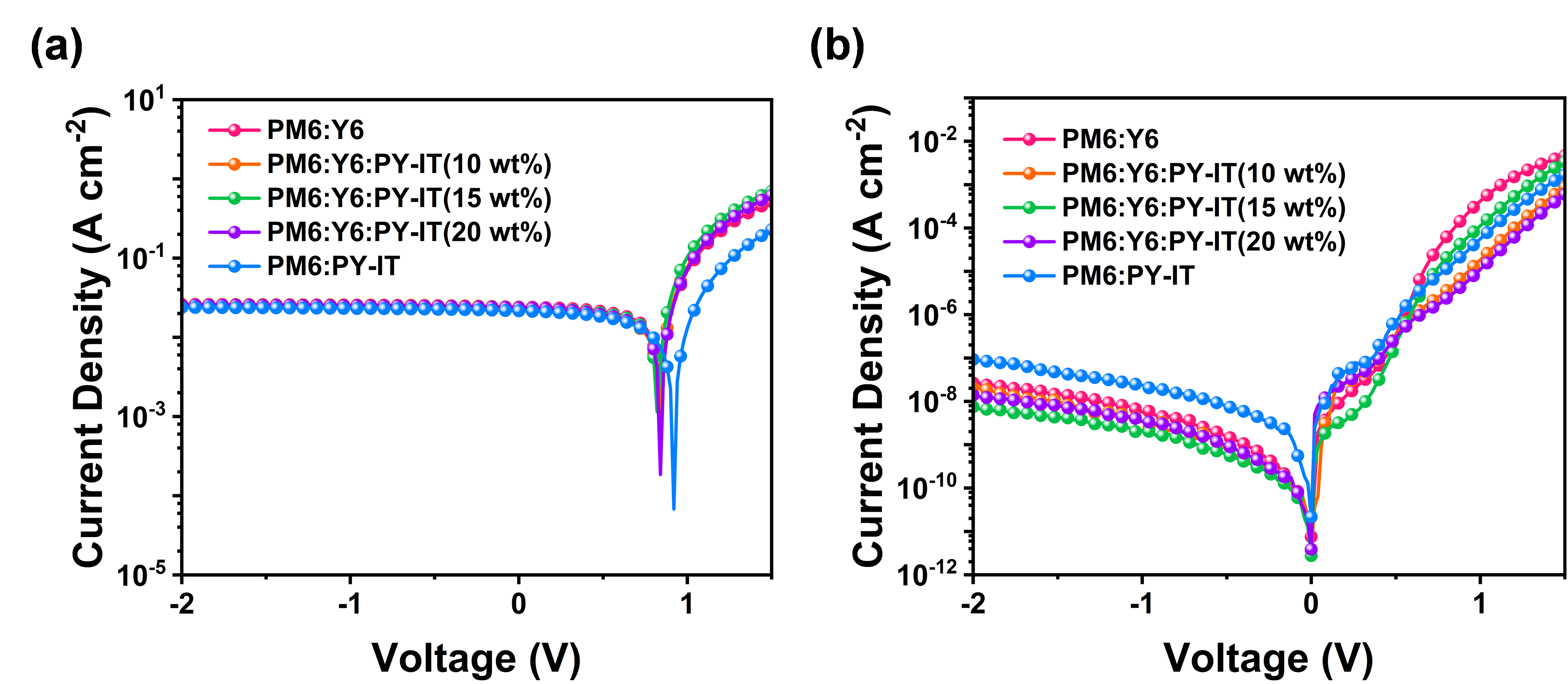


**Figure S7.** Current density–voltage (*J–V*) characteristics of PM6:Y6, PM6:PY-IT and Diluted devices with different amount of PY-IT under illumination AM 1.5G (a) and under dark conditions (b).


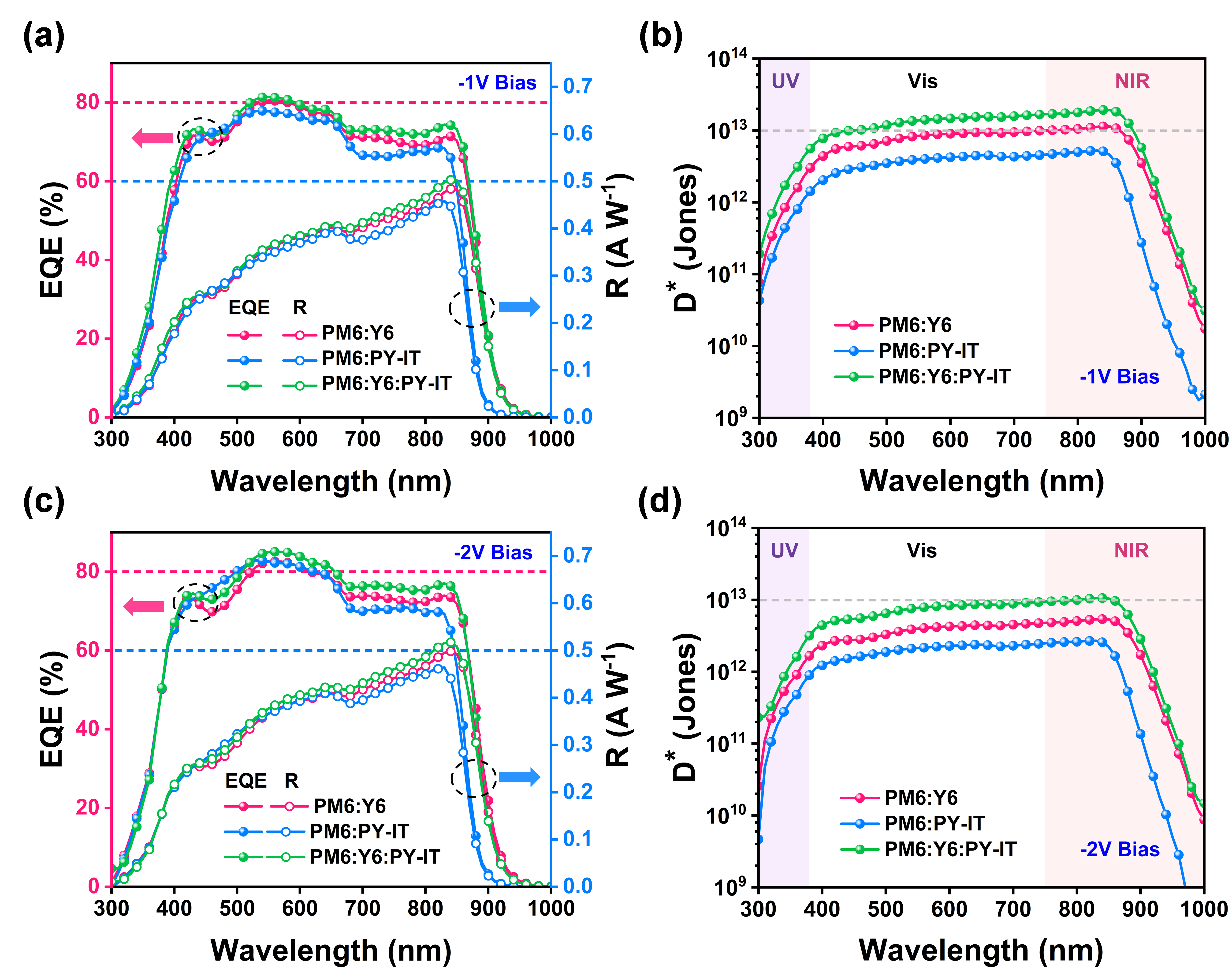


**Figure S8.** EQE, $R$ and *D^*^* of PM6:Y6, PM6:PY-IT and PM6:Y6:PY-IT(15 wt%) at -1 V and −2 V negative bias, respectively (a-d).


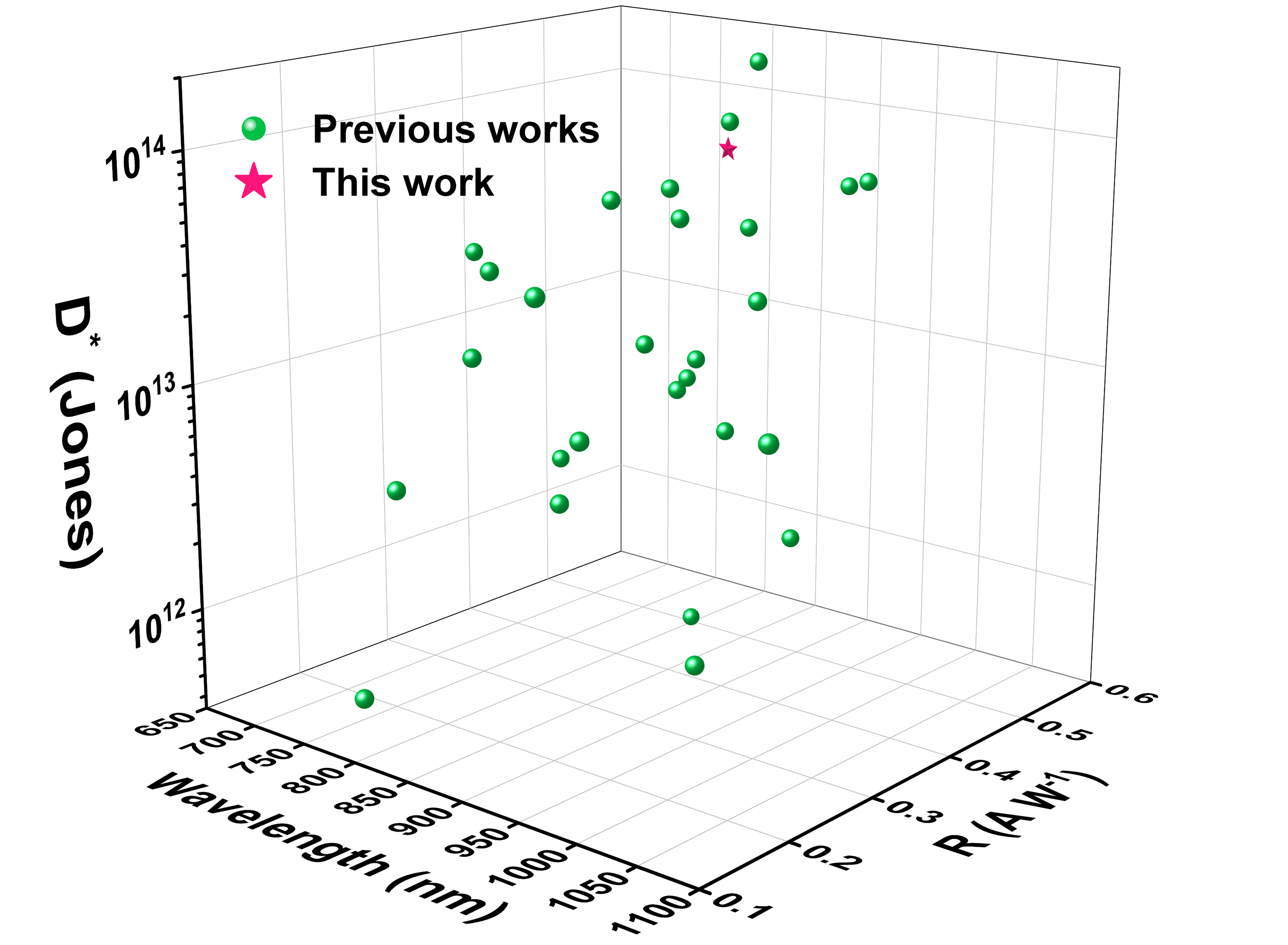


**Figure S9.** Comparison of the reported and our organic photodetectors.


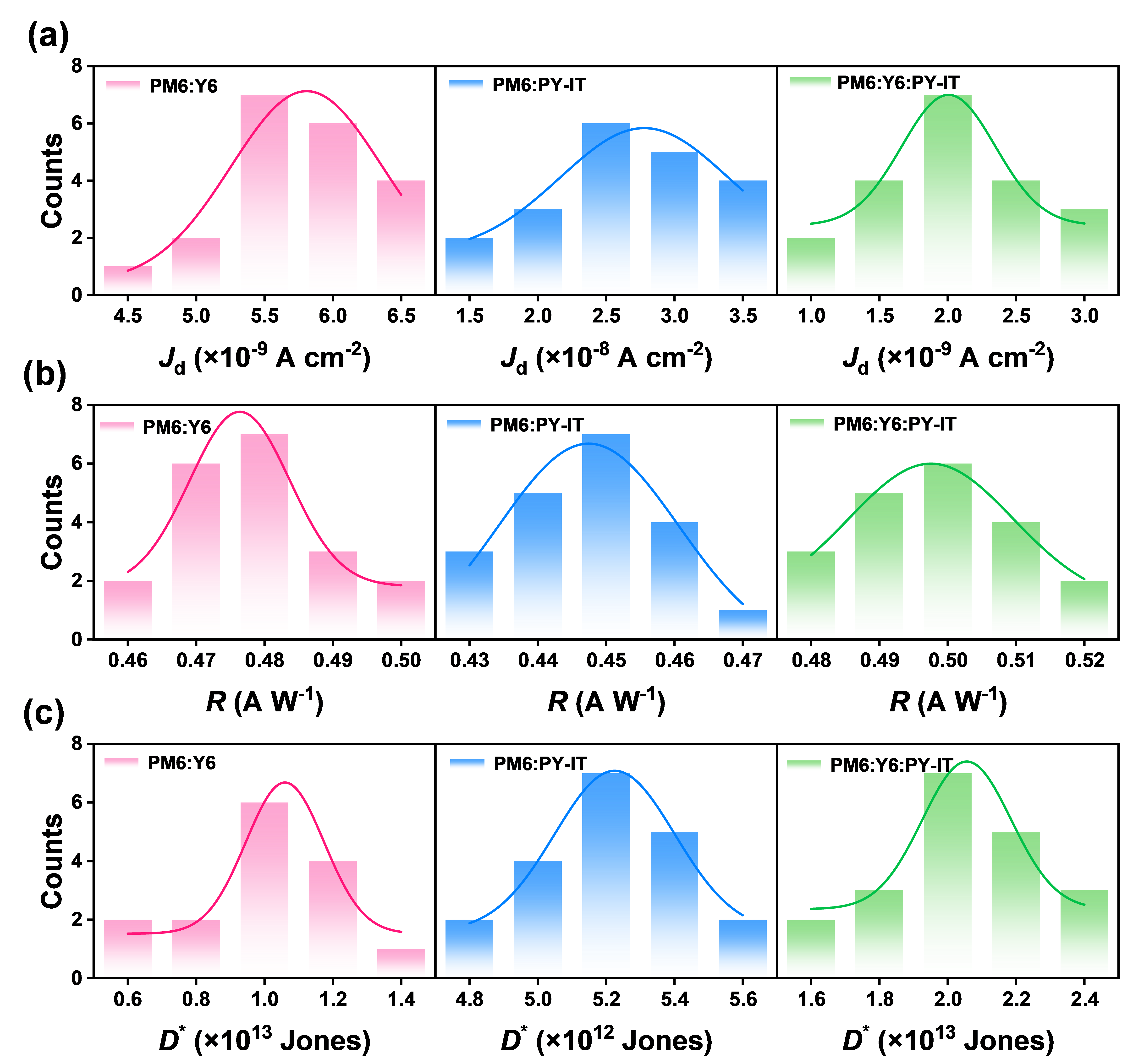


**Figure S10.** Statistical distributions of *J*_d_ (a), *R* (b), and *D^∗^* (c) for PM6:Y6, PM6:PY-IT and diluted devices at −1 V bias, histograms represent device counts per performance interval, and solid lines are Gaussian fits.


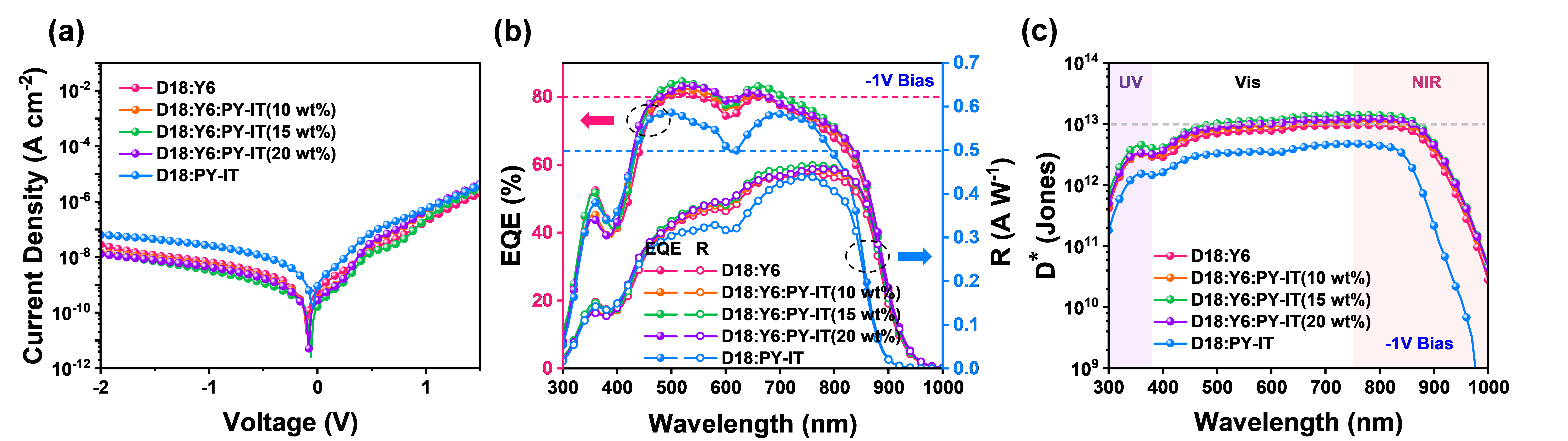


**Figure S11.** The *J-V* curves in dark (a), EQE and *R* curves (b), *D^*^* curves (c) of D18:Y6, D18:PY-IT and diluted devices.


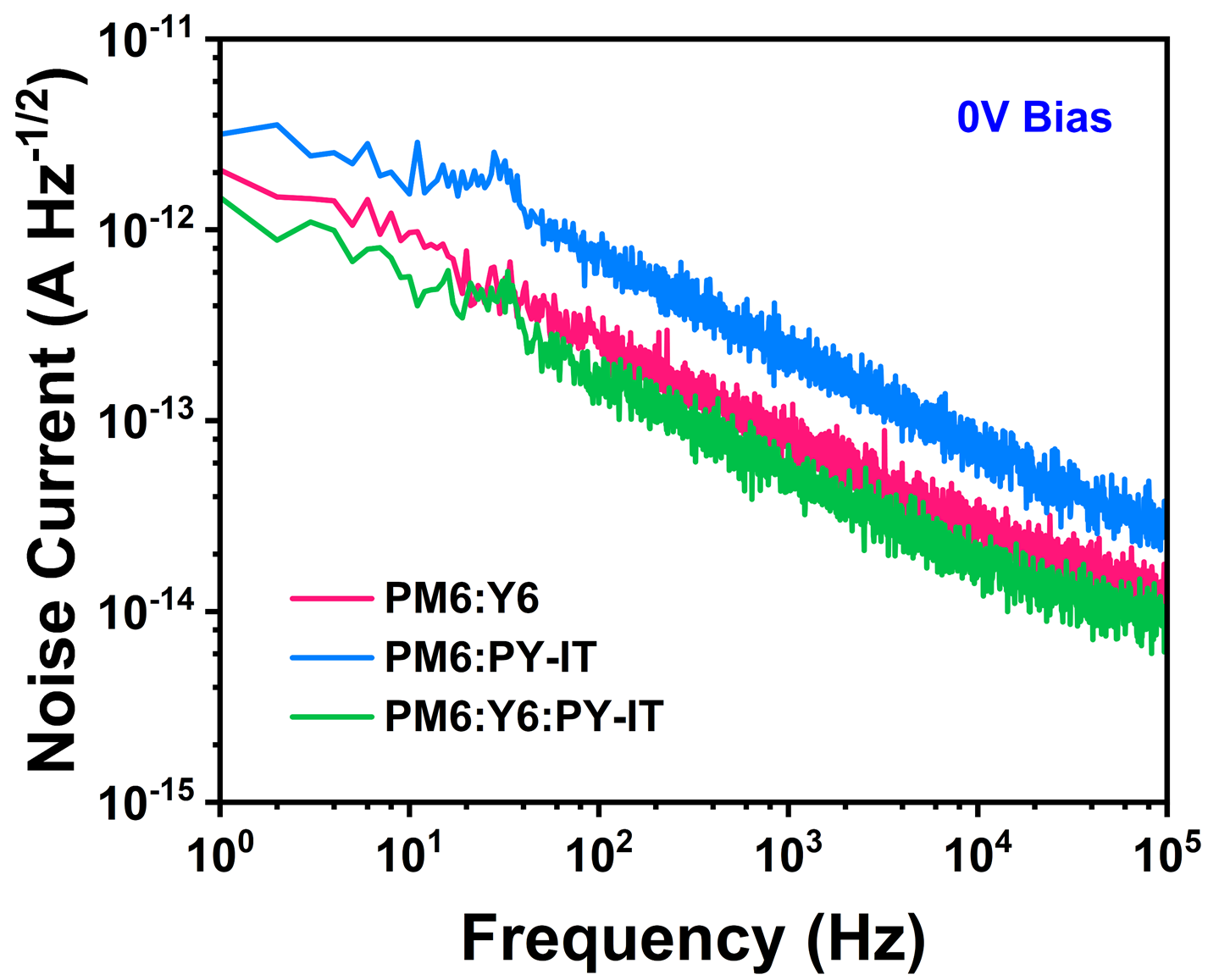


**Figure S12.** Noise current of PM6:Y6, PM6:PY-IT and PM6:Y6:PY-IT(15 wt%) at 0 V based OPDs.


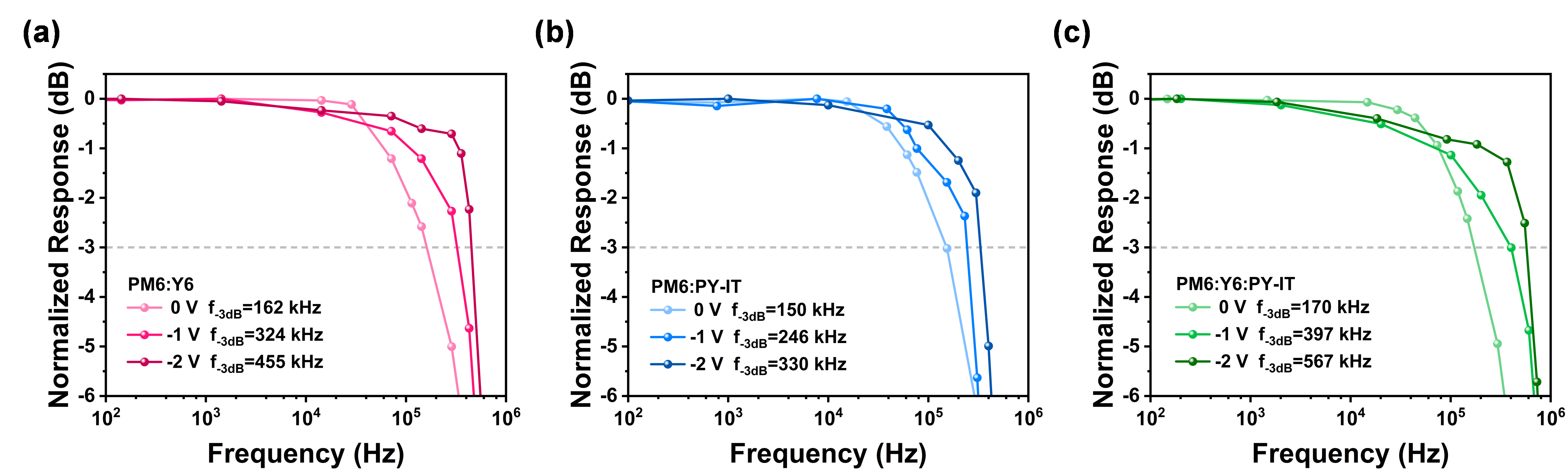


**Figure S13.** −3 dB cutoff frequency (a-c) of PM6:Y6, PM6:PY-IT and PM6:Y6:PY-IT(15 wt%) based OPDs at 0, −1 and −2 V negative bias, respectively.


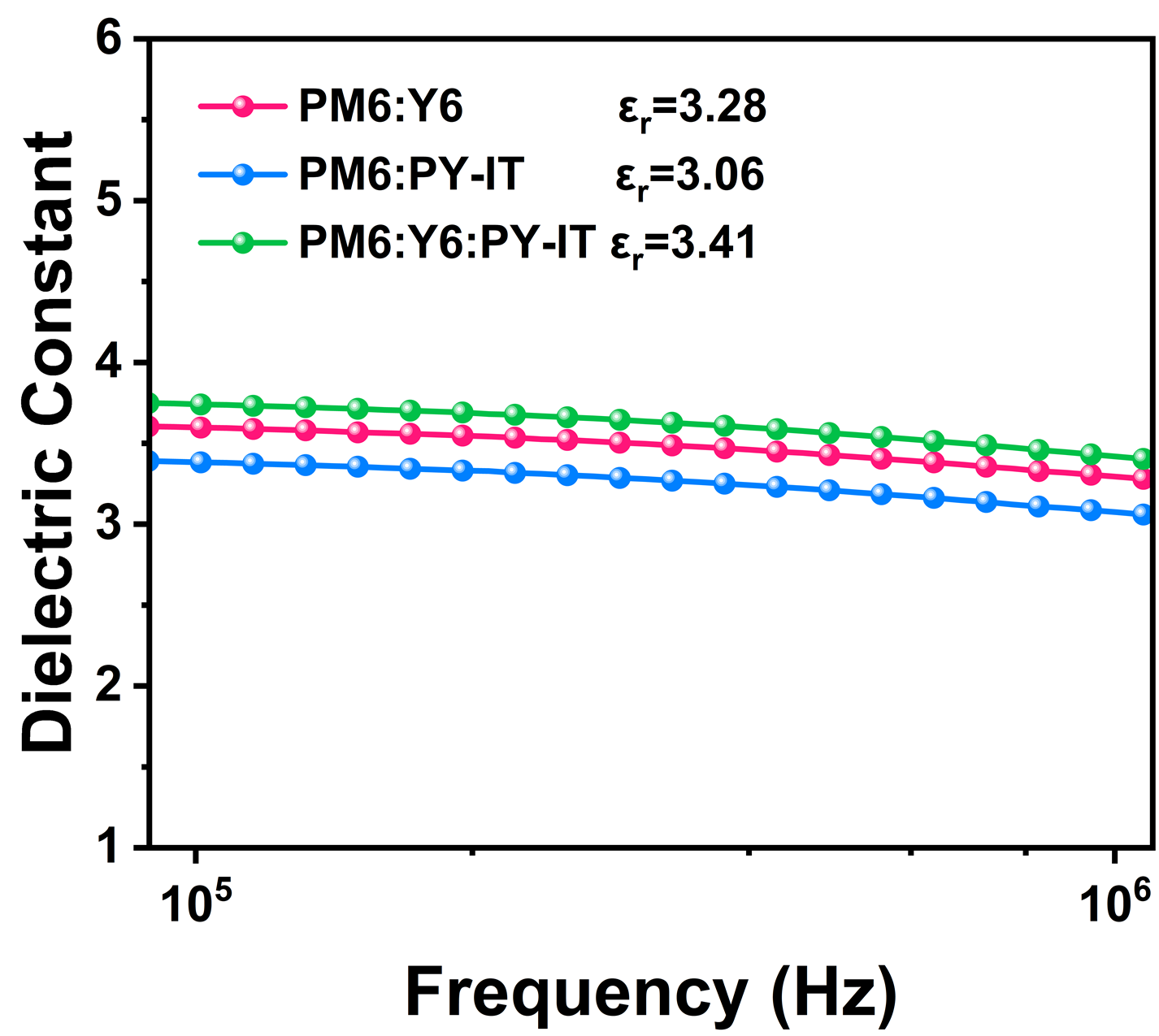


**Figure S14.** Relative dielectric constant of PM6:Y6, PM6:PY-IT and PM6:Y6:PY-IT(15 wt%).


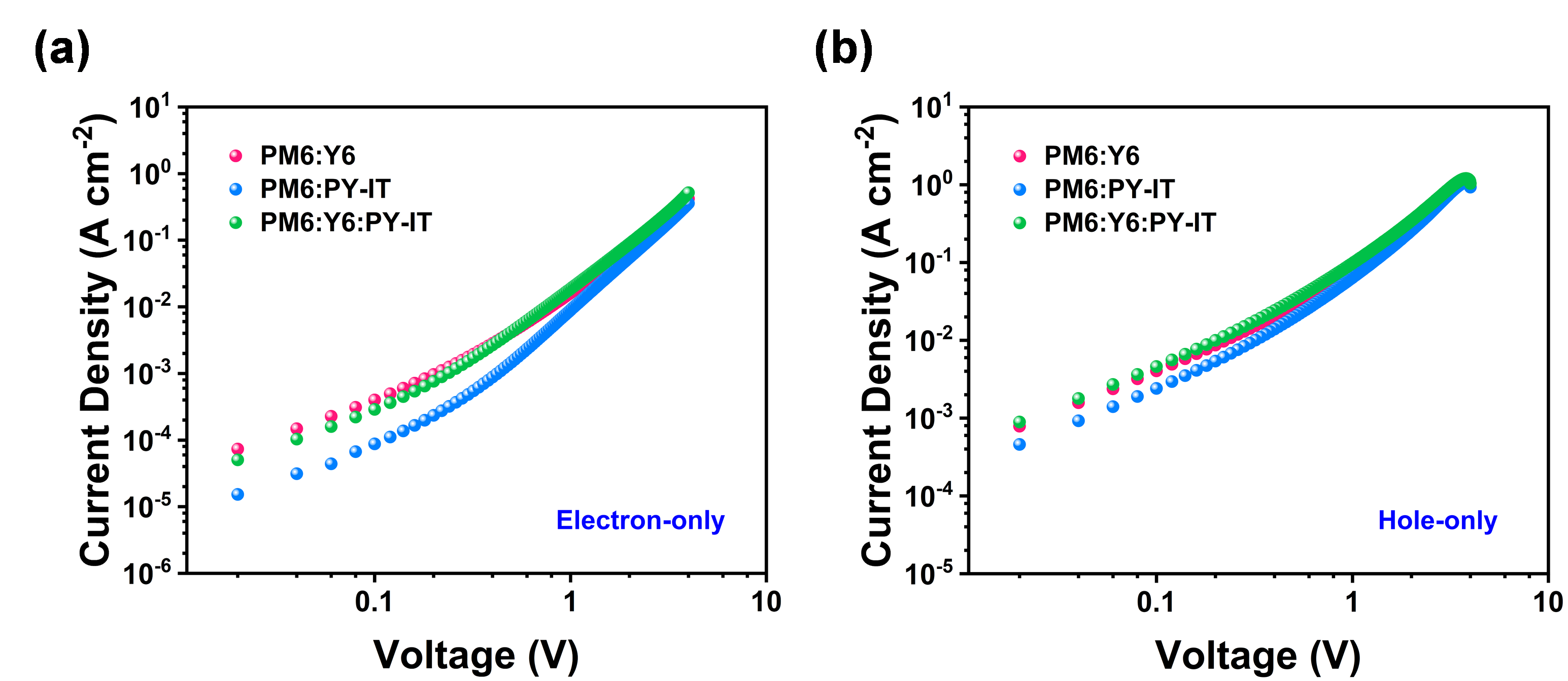


**Figure S15.** Electron mobility (a) and hole mobility (b) of PM6:Y6, PM6:PY-IT and PM6:Y6:PY-IT(15 wt%) measured by the SCLC method.


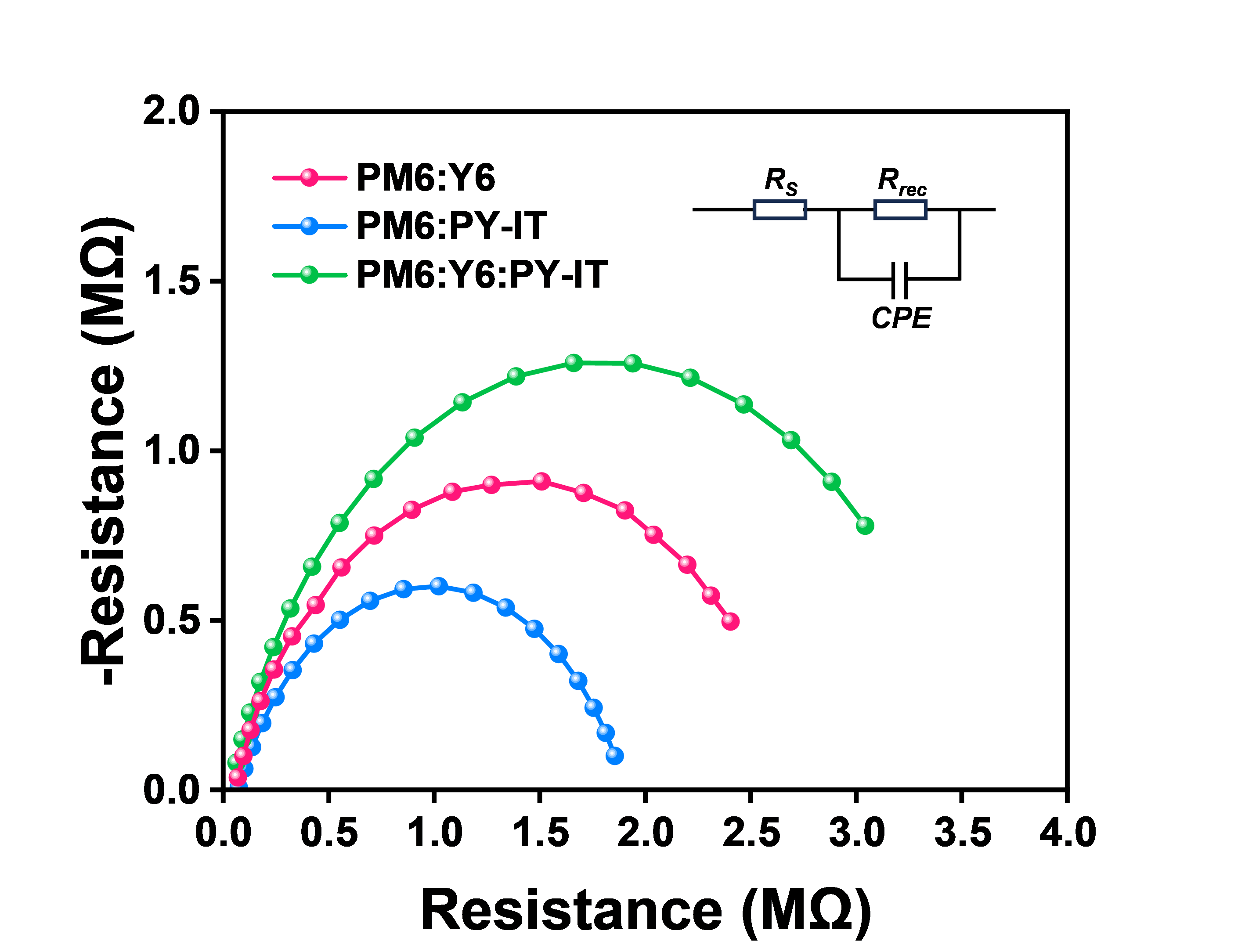


**Figure S16.** Nyquist curves of PM6:Y6, PM6:PY-IT and PM6:Y6:PY-IT(15 wt%) based blend films.


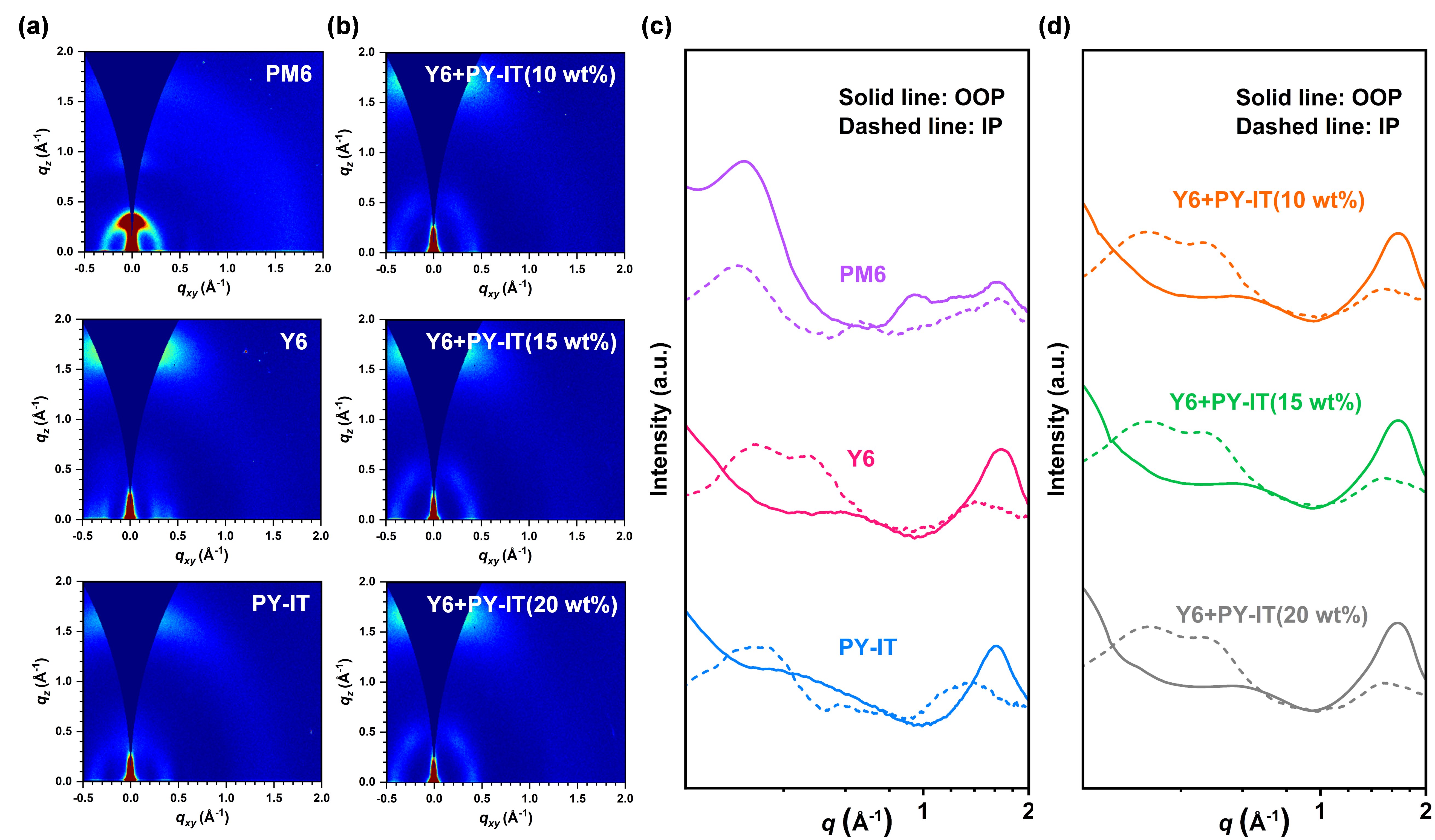


**Figure S17.** 2D GIWAXS patterns (a-b) and corresponding 1D line cuts (c-d) of PM6, Y6, PY-IT, Y6+PY-IT(10 wt%), Y6+PY-IT(15 wt%), Y6+PY-IT(20 wt%) based films.


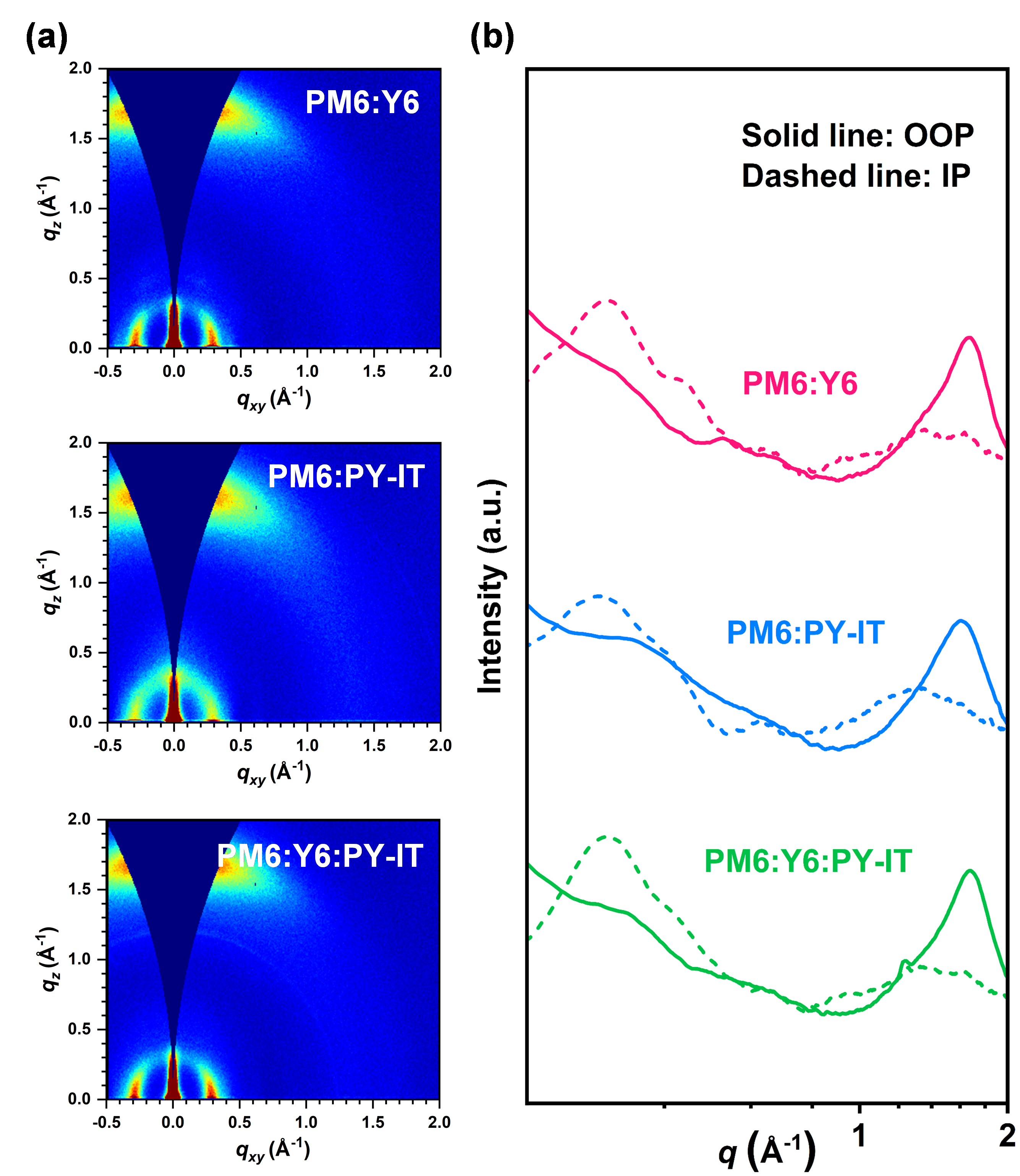


**Figure S18.** 2D GIWAXS patterns (a) and corresponding 1D line cuts (b) of PM6:Y6, PM6:PY-IT and PM6:Y6:PY-IT(15 wt%) based blend films.


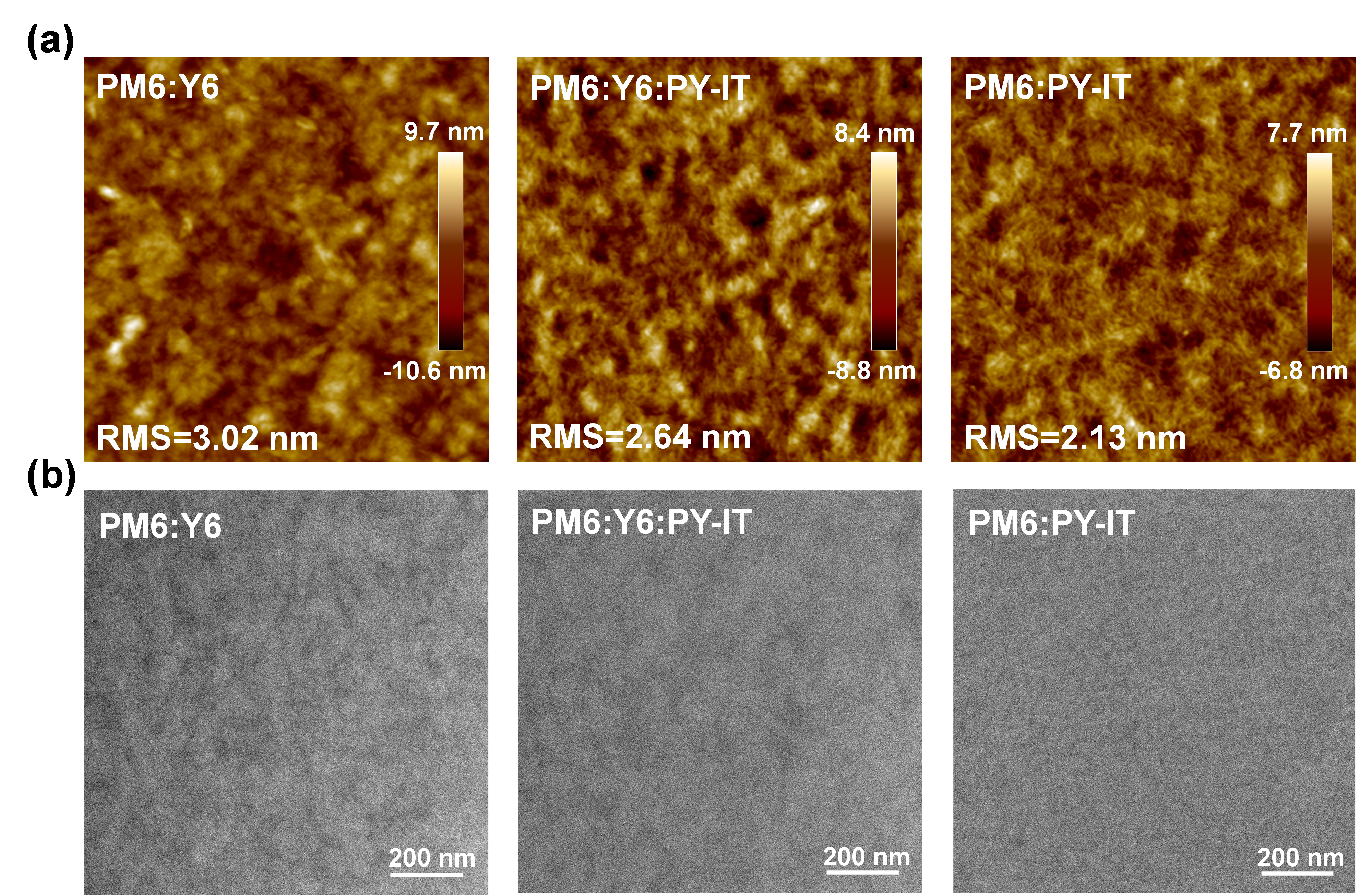


**Figure S19.** AFM height images (a), TEM images (b) of PM6:Y6, PM6:PY-IT and PM6:Y6:PY-IT(15 wt%) based blend films.


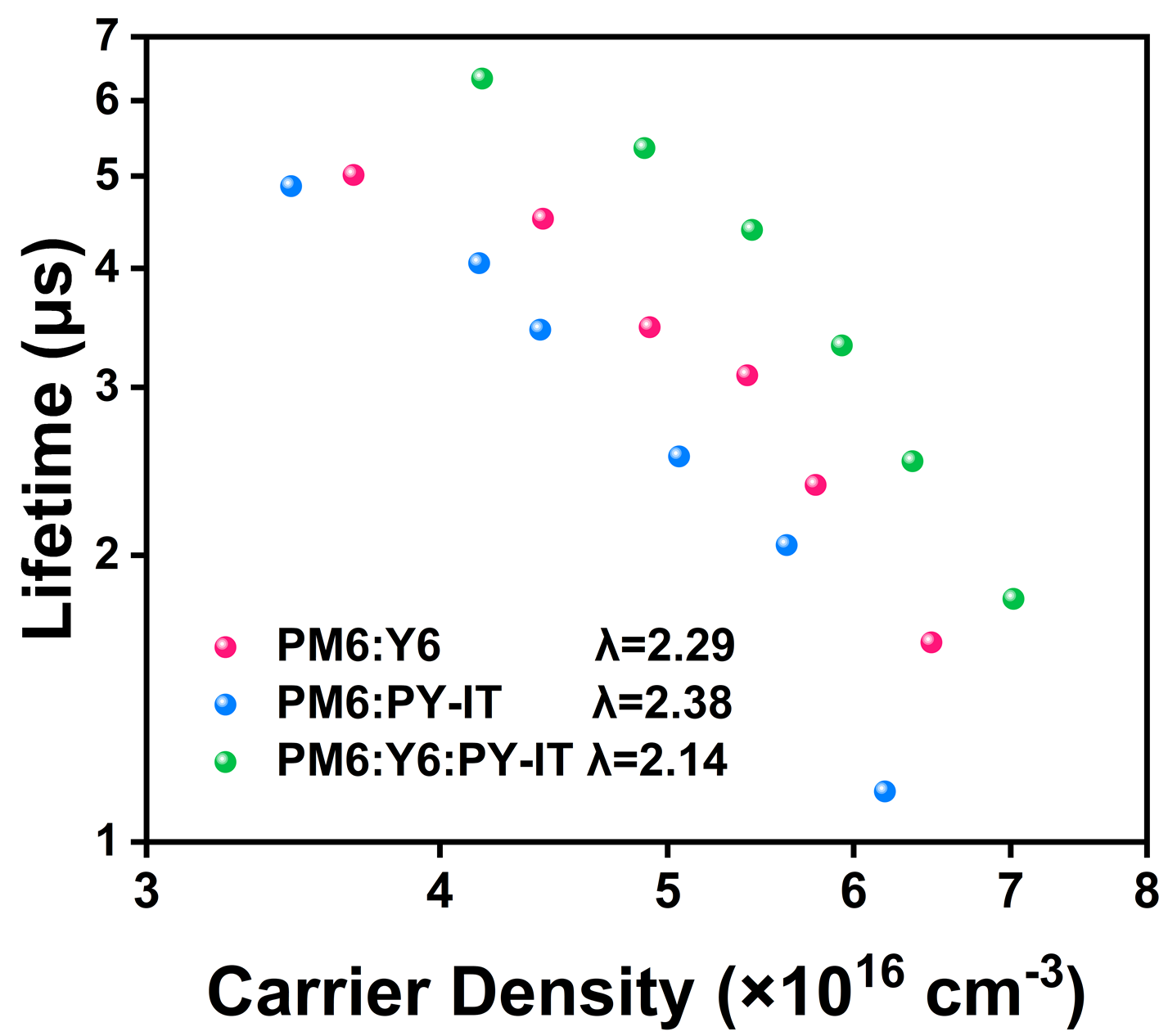


**Figure S20.** Carrier lifetime versus carrier density of PM6:Y6, PM6:PY-IT and PM6:Y6:PY-IT(15 wt%) devices.


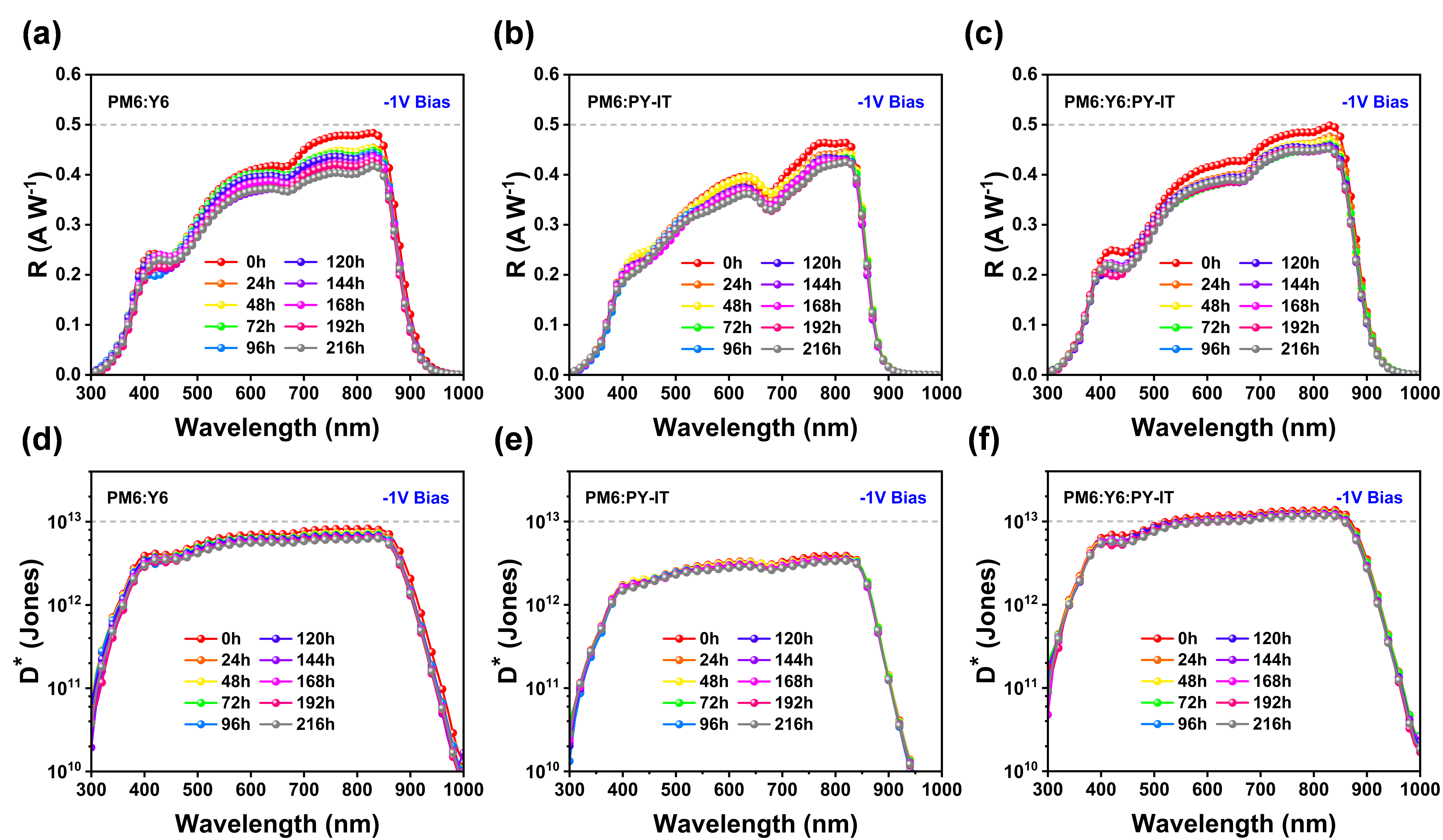


**Figure S21.** *R* (a-c) and *D*^*^ (d-f) curves of unencapsulated OPDs at −1 V after being stored in N_2_-filled glove box in the dark while heating at 85 °C.

**Table S1.** The optical and electrochemical properties of relevant pure materials.

| Material | $\lambda_{\max}^{\mathrm{film}}$ [nm] | $\lambda_{\mathrm{onset}}^{\mathrm{film}}$ [nm] | $E_{g}^{\mathrm{opt}}$^[a]^ [eV] |
| --- | --- | --- | --- |
| PM6 | 616 | 687 | 1.80 |
| Y6 | 827 | 920 | 1.35 |
| PY-IT | 815 | 887 | 1.40 |

^[a]^ Estimated from the onset absorption of thin films $E_{g}^{opt}$=1240/λ_onset_.

**Table S2.** The energy level of relevant pure materials.

| Material | *E*_HOMO_ [eV] | *E*_LUMO_ [eV] | $E_{g}$^[a]^ [eV] |
| --- | --- | --- | --- |
| PM6 | -5.44 | -3.55 | 1.89 |
| Y6 | -5.66 | -3.93 | 1.73 |
| PY-IT | -5.58 | -3.79 | 1.79 |

^[a]^ Calculated from the onsets of reduction/oxidation potentials.

**Table S3.** Contact angles, surface tension values and Flory–Huggins interaction parameters between donor and acceptor.

| Material | Contact angle [°] | | γ  [mN m^-1^] | χ_D:A_ | χ_A:A_ |
| --- | --- | --- | --- | --- | --- |
|  | H_2_O | CH_2_I_2_ |  |  |  |
| PM6 | 106.73 | 50.75 | 35.29 | / | / |
| PY-IT | 103.07 | 42.56 | 39.69 | 0.13 | 0.05 |
| Y6 | 97.86 | 35.64 | 42.56 | 0.34 | 0.05 |

**Table S4.** UPS-HOMO/LUMO estimated from UPS measurement.

| Material | *E*_HUMO_ [eV] | *E*_LOMO_ [eV] |
| --- | --- | --- |
| Y6 | -5.67 | -4.32 |
| Y6+PY-IT(5 wt%) | -5.65 | -4.30 |
| Y6+PY-IT(50 wt%) | -5.62 | -4.25 |
| Y6+PY-IT(95 wt%) | -5.60 | -4.21 |
| PY-IT | -5.59 | -4.19 |

**Table S5.** Summary of the photodetection performance of PM6:Y6, PM6:PY-IT and Diluted devices at −1 V and -2 V.

| Active layers | $J_{d}$ [A cm^-2^] [Bias] | $R_{\lambda max}$ [A W^-1^] [Bias] | $D_{\lambda max}^{*}$ [Jones] [Bias] |
| --- | --- | --- | --- |
| PM6: Y6 | 5.63 × 10^-9^ (-1 V)  2.65 × 10^-8^ (-2 V) | 0.484 (-1 V)  0.498 (-2 V) | 1.14 × 10^13^ (-1 V)  5.41 × 10^12^ (-2 V) |
| PM6: PY-IT | 2.37 ×10^-8^ (-1 V)  9.23 × 10^-8^ (-2 V) | 0.458 (-1 V)  0.464 (-2 V) | 5.26 × 10^12^ (-1 V)  2.70 × 10^12^ (-2 V) |
| PM6: Y6: PY-IT(15 wt%) | 2.13 × 10^-9^ (-1 V)  7.44 × 10^-9^ (-2 V) | 0.503 (-1 V)  0.517 (-2 V) | 1.93 × 10^13^ (-1 V)  1.06 × 10^13^ (-2 V) |

**Table S6.** Performance of NIR OPDs prepared in recent years.

| Photoactive layer | *J*_d_ [nA cm^-2^] | Bias [V] | $R$ [A W^-1^] | $D^{*}$ [Jones] | λ [nm] | Ref |
| --- | --- | --- | --- | --- | --- | --- |
| DHTBTEZP:PC_61_BM | 3.44 | 0 | 0.15 | 4.56 × 10^12^ (based on *J*_d_) | 800 | [1] |
| PbPc:C60 | / | 0 | 0.22 | 4.2 × 10^12^ (based on *J*_d_) | 890 | [2] |
| PDPP3T:PC_71_BM | 3.1 | -1 | 0.21 | 1.5 × 10^13^ (based on *J*_d_) | 820 | [3] |
| PMDPP3T:PC_61_BM | 3 | -0.2 | 0.37 | 1.23 × 10^13^ (based on *J*_d_) | 850 | [4] |
| PCE10: CTIC-4F | 0.89 | 0 | 0.46 | 1.5 × 10^12^ (based on *J*_d_) | 920 | [5] |
| D18:Y6 | / | -0.5 | 0.491 | 3.88 × 10^11^ (based on *J*_d_) | 805 | [6] |
| PCE10:Y6 | 11 | -0.5 | 0.41 | 6.9 × 10^12^ (based on *J*_d_) | 850 | [7] |
| P3HT:TPBT-OR | 35.7 | -0.5 | 0.084 | 7.87 × 10^11^ (based on *J*_d_) | ~826 | [8] |
| PNTB6-Cl:Y6 | 28.2 | / | 0.47 | 6.1 × 10^12^ (based on *S*_n_) | 815 | [9] |
| P3HT:IEICO4F:PC_71_BM | 247 | -0.1 | 0.41 | 1.35 × 10^12^ (based on *J*_d_) | / | [10] |
| CS-DP:PC_71_BM | 0.425 | 0 | 0.33 | 5.73 × 10^13^ (based on *J*_d_) | 850 | [11] |
| PCE10:IEICO-4F | 0.35 | -0.1 | 0.54 | 5.3 × 10^13^ (based on *J*_d_) | 930 | [12] |
| NT40:N2200 | 0.485 | -0.1 | 0.33 | 2.61 × 10^13^ (based on *J*_d_) | 720 | [13] |
| PCE10:BTPV-4F-eC9 | 0.465 | 0 | 0.56 | 4.6 × 10^13^ (based on *J*_d_) | 900 | [14] |
| PCE10:IEICO-4F | 0.15 | -0.3 | 0.43 | 4.58 × 10^12^ (based on *S*_n_) | 880 | [15] |
| NT812:IEICO-4F | 0.22 | -0.1 | 0.42 | 9.5 × 10^12^ (based on *S*_n_) | 860 | [16] |
| PBDB-T:FM2 | 0.201 | 0 | 0.372 | 4.65 × 10^13^ (based on *J*_d_) | 880 | [17] |
| PBDB-T:DO-4F | 0.86 | 0 | 0.50 | 3.05 × 10^13^ (based on *J*_d_) | 850 | [18] |
| PTB7-Th:P(NDI-T-TPD) | 0.23 | -0.1 | 0.22 | 2.6 × 10^13^ (based on *J*_d_) | / | [19] |
| PTB7-Th:COTCN2 | 108 | / | 0.23 | 1.18 × 10^12^ (based on *J*_d_) | 1000 | [20] |
| PTQ10:IDSe | 1.65 | / | 0.37 | 3 × 10^12^ (based on *S*_n_) | 770 | [21] |
| PBDB-T:PZF-V :PY-IT | 50 | -1 | 0.44 | 8.0 × 10^12^ (based on *J*_d_) | / | [22] |
| PCE10:BDP4Cl | 0.358 | / | 0.17 | 1.57 × 10^13^ (based on *J*_d_) | 1100 | [23] |
| PM6-Br50/Y6 | 0.403 | -0.5 | 0.486 | 4.27 ×10^13^ (based on *J*_d_) | / | [24] |
| PM6:BTP-eC9 (DIB) | 0.0635 | 0 | 0.46 | 1.0× 10^14^ (based on *J*_d_) | 860 | [25] |
| PTB7-Th:IEICO-4F | 0.21 | -2 | 0.40 | 5.6 × 10^13^ (based on *J*_d_) | 850 | [26] |
| PBPyT-EH:BTP-eC9 | 0.0287 | 0 | 0.51 | 1.67 × 10^14^ (based on *J*_d_) | 850 | [27] |
| PBDT-TT:DPPSe-4Cl:Y6 | 1 | -0.1 | 0.18 | 1.0 × 10^13^ (based on *J*_d_) | 940 | [28] |
| P4TOC-DCBT | / | 0 | 0.11 | 4.73 × 10^13^ (based on *J*_d_) | 960 | [29] |
| D18/Y18 | 0.395 | -2 | 0.48 | 4.24 × 10^13^ (based on *J*_d_) | / | [30] |
| PM6:CT-Cl | 0.71 | -1 | 0.37 | 2.4 × 10^13^ (based on *J*_d_) | 950 | [31] |
| PBDB-T:TBT-V-Cl | 0.669 | 0 | 0.23 | 3.33 × 10^13^ (based on *J*_d_) | 820 | [32] |
| PM6:Y6:PY-IT | 0.152 | -0.2 | 0.486 | 6.98 × 10^13^ (based on *J*_d_) | 840 | This work |

**Table S7.** Summary of the photodetection performance of D18: Y6, D18: PY-IT and diluted devices at −1 V.

| Active layers | $J_{d}$ [A cm^-2^] [Bias] | $R_{\lambda max}$ [A W^-1^] [Bias] | $D_{\lambda max}^{*}$ [Jones] [Bias] |
| --- | --- | --- | --- |
| D18: Y6 | 6.69 × 10^-9^ | 0.446 | 9.63 × 10^12^ |
| D18: Y6: PY-IT(10 wt%) | 5.17 × 10^-9^ | 0.454 | 1.12 × 10^13^ |
| D18: Y6: PY-IT(15 wt%) | 3.41 × 10^-9^ | 0.465 | 1.41 × 10^13^ |
| D18: Y6: PY-IT(20 wt%) | 4.45 × 10^-9^ | 0.457 | 1.21 × 10^13^ |
| D18: PY-IT | 2.65 × 10^-8^ | 0.440 | 4.78 × 10^12^ |

**Table S8.** Summary of the fitting data for hole-only and electron-only devices.

| Active layer | *μ*_h_ [10^−4^ cm^2^ V^−1^ s^−1^] | *μ*_e_ [10^−4^ cm^2^ V^−1^ s^−1^] | *μ*_h_/*μ*_e_ |
| --- | --- | --- | --- |
| PM6:Y6 | 2.65 | 1.95 | 1.36 |
| PM6:PY-IT | 2.55 | 1.77 | 1.44 |
| PM6:Y6:PY-IT(15 wt%) | 2.91 | 2.84 | 1.02 |

**Table S9.** Summary of the parameters for the pure and blend films calculated from GIWAXS profiles.

| Film | Out-of-plane (010) | | | In-plane (100) | | |
| --- | --- | --- | --- | --- | --- | --- |
|  | *q*  [Å^-1^] | *d*-spacing  [Å] | CCL  [Å] | *q*  [Å^-1^] | *d*-spacing  [Å] | CCL  [Å] |
| PM6 | 1.638 | 3.83 | 23.32 | 0.301 | 20.86 | 61.42 |
| Y6 | 1.681 | 3.74 | 22.36 | 0.342 | 18.36 | 33.52 |
| PY-IT | 1.632 | 3.85 | 22.94 | 0.330 | 19.03 | 32.56 |
| Y6+PY-IT(10 wt%) | 1.681 | 3.74 | 22.27 | 0.341 | 18.42 | 33.46 |
| Y6+PY-IT(15 wt%) | 1.682 | 3.73 | 22.14 | 0.341 | 18.42 | 33.31 |
| Y6+PY-IT(20 wt%) | 1.682 | 3.73 | 22.09 | 0.340 | 18.47 | 33.15 |
| PM6:Y6 | 1.672 | 3.76 | 23.58 | 0.302 | 20.80 | 72.18 |
| PM6:PY-IT | 1.612 | 3.90 | 21.15 | 0.294 | 21.36 | 62.52 |
| PM6:Y6:PY-IT(15 wt%) | 1.681 | 3.74 | 23.21 | 0.303 | 20.73 | 69.86 |

**Table S10.** Summary of thermal stability related data of unencapsulated OPDs at -1 V.

| Active layers | Before heating | | After heating 9 days | | Attenuation percentage [%] | |
| --- | --- | --- | --- | --- | --- | --- |
|  | $R_{\lambda max}$  [A W^-1^]  (-1V bias) | $D_{\lambda max}^{*}$  [Jones]  (-1V bias) | $R_{\lambda max}$  [A W^-1^]  (-1V bias) | $D_{\lambda max}^{*}$  [Jones]  (-1V bias) | $R_{\lambda max}$  [A W^-1^]  (-1V bias) | $D_{\lambda max}^{*}$  [Jones]  (-1V bias) |
| PM6: Y6 | 0.483 | 8.16 × 10^12^ | 0.418 | 6.31 × 10^12^ | 86.54 % | 77.33 % |
| PM6: PY-IT | 0.464 | 3.88 × 10^12^ | 0.425 | 3.36 × 10^12^ | 91.59 % | 86.59 % |
| PM6: Y6: PY-IT(15 wt%) | 0.498 | 1.37 × 10^13^ | 0.452 | 1.17 × 10^13^ | 90.76 % | 85.40 % |

**Supplementary references**

[1] L. Li, Y. Huang, J. Peng, Y. Cao, X. Peng, *J. Mater. Chem. C* **2014**, *2*, 1372.

[2] Z. Su, F. Hou, X. Wang, Y. Gao, F. Jin, G. Zhang, Y. Li, L. Zhang, B. Chu, W. Li, *ACS Appl. Mater. Interfaces* **2015**, *7*, 2529.

[3] X. Zhou, D. Yang, D. Ma, *Adv. Opt. Mater*. **2015**, *3*, 1570.

[4] S. Xiong, J. Tong, L. Mao, Z. Li, F. Qin, F. Jiang, W. Meng, T. Liu, W. Li, Y. Zhou, *J. Mater. Chem. C* **2016**, *4*, 1414.

[5] J. Lee, S.-J. Ko, H. Lee, J. Huang, Z. Zhu, M. Seifrid, J. Vollbrecht, V. V. Brus, A. Karki, H. Wang, K. Cho, T.-Q. Nguyen, G. C. Bazan, *ACS Energy Lett*. **2019**, *4*, 1401.

[6] Y. Wei, H. Chen, T. Liu, S. Wang, Y. Jiang, Y. Song, J. Zhang, X. Zhang, G. Lu, F. Huang, Z. Wei, H. Huang, *Adv. Funct. Mater*. **2021**, *31*, 2106326.

[7] J. Y. Son, H. J. Eun, G. U. Seo, A. Y. Lee, J.-H. Kim, J. H. Kim, *Dyes Pigm*. **2023**, *209*, 110907.

[8] S. Zhong, H. Chen, J. Yi, T. Yang, Z. Gan, X. Su, M. Li, Z. Zhong, F. Peng, L. Ying, *Org. Electron*. **2022**, *109*, 106610.

[9] Y. Gao, J. Liao, H. Chen, H. Ning, Q. Wu, Z. Li, Z. Wang, X. Zhang, M. Shao, Y. Yu, *Adv. Sci*. **2022**, *10*, 2204727.

[10] Y. Han, X. Zhang, F. Zhang, D. Zhao, J. Yu, *Org. Electron*. **2019**, *75*, 105410.

[11] L. Xiao, S. Chen, X. Chen, X. Peng, Y. Cao, X. Zhu, *J. Mater. Chem. C* **2018**, *6*, 3341.

[12] Y. Song, G. Yu, B. Xie, K. Zhang, F. Huang, *Appl. Phys. Lett*. **2020**, *117*, 093302.

[13] Z. Zhong, K. Li, J. Zhang, L. Ying, R. Xie, G. Yu, F. Huang, Y. Cao, *ACS Appl. Mater. Interfaces* **2019***, 11*, 14208.

[14] T. Liu, Z. Jia, Y. Song, N. Yu, Q. Lin, C. Li, Y. Jia, H. Chen, S. Wang, Y. Wei, Y. Lin, F. Huang, Z. Tang, Y. Li, L. Meng, H. Huang, *Adv. Funct. Mater.* **2023**, *33*, 2301167.

[15] T. Liu, Q. Lin, Y. Ma, S. Wang, H. Chen, Y. Wei, Y. Song, L. Shen, F. Huang, H. Huang, *Adv. Opt. Mater*. **2022**, *10*, 2201104.

[16] H. Quan, Z. Zhong, T. Hao, K. An, W. Zhong, C. Wang, F. Liu, L. Ying, F. Huang, *Chem. Eng. J*. **2023**, *452*, 139295.

[17] Y. Zhang, Y. Yu, X. Liu, J. Miao, Y. Han, J. Liu, L. Wang, *Adv. Mater*. **2023**, *35*, 2211714.

[18] Y. Xu, T. Zhang, H. Yao, J. Wang, P. Bi, J. Hou, *J. Energy Chem*. **2022**, *72*, 388.

[19] X. Wang, Z. Y. Wang, S. Gao, J. Han, Y. Xu, Z. Liu, H. Wang, Y. Zhang, W. Qiao, *Chem. Eng. J*. **2023**, *474*, 145529.

[20] J. W. Ha, H. J. Eun, B. Park, H. Ahn, D. R. Hwang, Y. S. Shim, J. Heo, C. Lee, S. C. Yoon, J. H. Kim, S. J. Ko, *Adv. Funct. Mater*. **2022**, *33*, 2211486.

[21] Z. Qiao, Q. He, A. D. Scaccabarozzi, J. Panidi, A. Marsh, Y. Han, P. Jacoutot, D. Nodari, T. Zhang, A. Way, A. J. P. White, T. D. Anthopoulos, W. C. Tsoi, A. A. Bakulin, M. Heeney, Z. Fei, N. Gasparini, *J. Mater. Chem. C* **2024**, *12*, 5766.

[22] H. T. Chandran, R. Ma, Z. Xu, J. C. Veetil, Y. Luo, T. A. Dela Peña, I. Gunasekaran, S. Mahadevan, K. Liu, Y. Xiao, H. Xia, J. Wu, M. Li, S. W. Tsang, X. Yu, W. Chen, G. Li, *Adv. Mater*. **2024**, *36*, 2407271.

[23] M. Yang, B. Yin, G. Hu, Y. Cao, S. Lu, Y. Chen, Y. He, X. Yang, B. Huang, J. Li, B. Wu, S. Pang, L. Shen, Y. Liang, H. Wu, L. Lan, G. Yu, F. Huang, Y. Cao, C. Duan, *Chem* **2024**, *10*, 1425.

[24] M. Hun Jee, B. Park, A. Young Lee, S. Rhee, M. Lim, J. Min Ha, N. Kim, F. Zhang, J. W. Ha, H. Ahn, J. H. Kim, M. Han, S. Cheol Yoon, S.-J. Ko, H. Y. Woo, *Chem. Eng. J.* **2024**, 490, 151624.

[25] J. W. Qiao, F. Z. Cui, W. Q. Zhang, R. H. Gui, Z. Fu, M. Sun, P. Lu, H. Yin, X. Y. Du, X. T. Hao, *Adv. Mater*. **2025**, *37*, 2418844.

[26] W. Yang, W. Qiu, E. Georgitzikis, E. Simoen, J. Serron, J. Lee, I. Lieberman, D. Cheyns, P. Malinowski, J. Genoe, H. Chen, P. Heremans, *ACS Appl. Mater. Interfaces* **2021**, *13*, 16766.

[27] T. Liu, T. Wang, F. Bi, S. Yang, Y. Zhao, B. Tang, J. Wang, Y. Li, J. Chu, X. Bao, *Adv. Funct. Mater*. **2025**, e16379.

[28] Y. Wang, M. Yang, B. Yin, B. Wu, G. Liu, S. Jeong, Y. Zhang, C. Yang, Z. He, F. Huang, Y. Cao, C. Duan, *ACS Appl. Mater. Interfaces* **2024**, *16*, 66846.

[29] X. Chen, Y. Zhu, Y. Xu, M. Rao, P. Pang, B. Zhang, C. Xu, W. Ni, G. Li, J. Wu, M. Li, Y. Chen, Y. Geng, *Angew. Chem. Int. Ed*. **2024**, *64*, e202413965.

[30] B. H. Jiang, Y. Y. Hsu, Y. W. Su, S. F. Peng, Z. E. Shi, C. P. Chen, *Adv. Opt. Mater.* **2025**, *13*, 2500258.

[31] K. H. Huang, B. H. Jiang, H. C. Lu, Y. J. Xue, C. F. Lu, Y. Y. Chang, C. L. Huang, S. Y. Chien, C. P. Chen, Y. J. Cheng, *Adv Sci.* **2025**, *12*, 2413045.

[32] W. Liu, W. Guo, L. Fu, Y. Duan, G. Han, J. Gao, H. Liu, Y. Wang, Z. Ma, Y. Liu, *Angew. Chem. Int. Ed*. **2024**, *64*, e202416751.
